# Multi-locus investigation of *Anopheles*-mediated selective pressure on *Plasmodium falciparum* in Africa

**DOI:** 10.1186/s13071-024-06604-y

**Published:** 2024-12-23

**Authors:** Isuru Gunarathna, Joseph D. Spear, Tamar E. Carter

**Affiliations:** Department of Biology, College of Arts and Sciences, Baylor University, Waco, TX USA

**Keywords:** Vector–parasite interactions, Vector-mediated selection, Genetics, Coevolution

## Abstract

**Background:**

The high burden of malaria in Africa is largely due to the presence of competent and adapted *Anopheles* vector species. With invasive *Anopheles stephensi* implicated in malaria outbreaks in Africa, understanding the genomic basis of vector-parasite compatibility is essential for assessing the risk of future outbreaks due to this mosquito. Vector compatibility with *P. falciparum* arises from ancient coevolution and involves genes such as *Pfs47* in *P. falciparum* and *P47Rec* in *Anopheles*. Questions remain about whether sub-continental vector variation is a selective pressure on current *Plasmodium* populations.

**Methods:**

We analyzed the genetic diversity in parasite–vector interaction genes in *P. falciparum* and *An. gambiae* from 9 and 15 countries in Africa, respectively. Specifically, we looked for evidence of malaria vector-mediated selection within three *P. falciparum* genes (*Pfs47*, *Pfs16*, *Pfs37*) and conducted association analyses with occurrence probabilities of prominent malaria vectors.

**Results:**

Higher protein haplotype diversities of *Pfs47* and *Pfs16* were associated with the probability of occurrence of *An. arabiensis* and *An. funestus* together. Only *Pfs16* carried a signature of positive selection consistently (average Tajima’s *D* = −2.96), which was associated with the probability of occurrence of *An. funestus*. These findings support vector-mediated selection on the basis of vector species diversity that may be occurring within Africa. We also employed phylogenetic analyses of *An. gambiae* interaction genes (*P47Rec*, *APN1*, *HPX15*) to identify significant subspecies diversity as a prerequisite to vector-population-mediated selection. *Anopheles gambiae* *HPX15* revealed significant within-species differentiation (multiple branches bootstrap > 70) compared with absence of variation in *P47Rec*, suggesting that further investigation into subspecies-mediated selection on the basis of *HPX15* is needed. Finally, we observed five amino acid changes at *P47Rec* in invasive *An. stephensi* compared with dominant African *Anopheles* species, calling for further investigation of the impact these distinct *P47Rec* variants might have on local African *P. falciparum* *Pfs47* diversity.

**Conclusions:**

Overall, these findings suggest that vector variation within Africa could influence *P. falciparum* diversity and lay a genomic framework for future investigation of invasive *An. stephensi*’s impact on African malaria.

**Graphical Abstract:**

**Figure:**
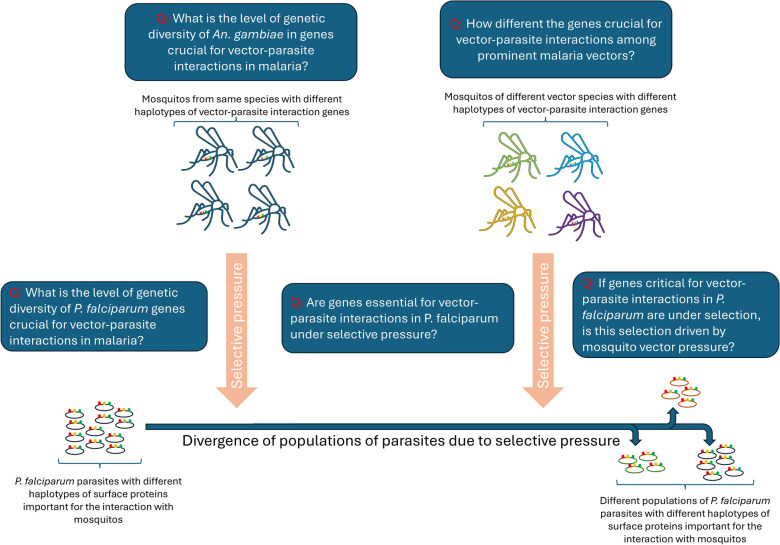

**Supplementary Information:**

The online version contains supplementary material available at 10.1186/s13071-024-06604-y.

## Background

In 2022, more than 249 million cases of malaria were reported, with the majority located in Africa [1]. Efforts to control this disease, which impacts half of the world’s population, have reached a critical point in the last few years following the 10% increase in cases observed in 2020 [2]. Most of these cases are due to the unicellular eukaryotic parasite *Plasmodium falciparum* adapted to spread through *Anopheles gambiae *s.l. mosquitoes present throughout most of the African continent [3]. Most recently, *Anopheles stephensi*, common to South Asia and the Middle East, has invaded the Horn of Africa and several other African countries, further exasperating malaria control [4–6]. Vectorial competence, breeding habitats, and behaviors vary even among the members of the native *An. gambiae* s.l. complex [7]. With the invasion of *An. stephensi* now contributing to increased complexity of the already diverse vector species composition in Africa, it is important to determine the new transmission dynamics in *An. stephensi*-invaded areas.

Interactions between *Plasmodium* and the mosquito midgut serve as the critical gateway for malaria transmission. The parasite invasion of the mosquito midgut requires an interaction between both parasite and mosquito proteins [8]. Previous studies have shown that the level of compatibility of interacting proteins between malaria vector species and parasite species varies depending on the haplotypes of the genes coding for these proteins, especially during the midgut invasion [9]. Many of the *Anopheles* and *Plasmodium* genes responsible for these interactions are currently being studied for their potential use in development of transmission blocking vaccines [10, 11].

In addition to within-species genetic diversity, the haplotype diversity of genes involved in interactions with malaria parasites across different vector species may influence the genetic diversity of key genes that mediate vector–parasite interactions in *Plasmodium falciparum*. Different mosquito species, such as *An. gambiae* and *An. funestus*, have varying ecological niches, behaviors, and interactions with the malaria parasite, which can lead to differential selective pressures on the parasite’s genes [12]. For instance, variations in the vector’s immune response, feeding habits, and geographical distribution can drive genetic diversity in the parasite as it adapts to survive and thrive in different vector environments [13]. Consequently, regions with diverse vector species compositions are likely to exhibit higher nucleotide and haplotype diversity in *P. falciparum* genes associated with vector interactions, reflecting the parasite’s adaptation to a range of vector-related selective pressures.

A well-studied example for parasite–vector interaction genes is the *Pfs47*-*P47Rec* complex in *P. falciparum* and *An. gambiae* [11, 14, 15]. The mosquito midgut protein *P47Rec* and parasite protein *Pfs47* work as a receptor–ligand pair during the *Plasmodium* invasion by playing a role in the immune evasion of parasites to make the parasite “undetectable” to the mosquito immune system. Silencing *P47Rec* expression has reduced the infection of *P. falciparum* in *An. gambiae* mosquitoes [16].

With this “lock-and-key” type mechanism, the ability of *P. falciparum* strains to invade the *Anopheles* midgut cells is dependent on the correct matching of *Pfs47* surface protein haplotype (“the key”) with the *Anopheles* midgut receptor *P47Rec* (“the lock”) [16]. Previous functional studies demonstrated that replacing *Pfs47* haplotype in African *P. falciparum* with a different haplotype from another continent is sufficient to change the compatibility between the vector and parasite [9]. Later studies have shown that *Pfs47* is important for the adaptation of *P. falciparum* to different malaria vectors in different continents [17]. This vector-mediated selective pressure at the continental level in *Pfs47* resulted in significant population structure between different continents, particularly in domain 2 of the protein [14, 18, 19]. Subcontinental selective pressure on the *Pfs47* has been observed in previous studies in Nigeria, Brazil, and Malaysia [19]. Still, significant knowledge gaps remain about the level of vector-mediated selective pressure on *Pfs47* at a subcontinental level in Africa. This is important to evaluate given the multiple *Anopheles* vector species that exist sympatrically across Africa, some quite divergent from one another (e.g., the *An. gambiae* complex versus *Anopheles funestus*) [20].

In addition to the *Pfs47*-*P47Rec* system, there are several other protein coding genes being studied as transmission blocking vaccine (TBV) targets on the basis of their role in parasite–vector compatibility. Therefore, like *Pfs47* and *P47Rec*, these parasite and vector genes may also involve vector-mediated selection [10, 15]. In our study we selected two more genes from *P. falciparum* and another two genes from *An. gambiae*, which are important for vector–parasite interactions. In *Plasmodium* parasites, *Pfs16* (PF3D7_0406200) and *Pfs37* (PF3D7_1204400) have been recognized to be important for vector–parasite interactions because of their significant upregulated expression in the sexual stages and interactions with mosquito midgut proteins [15]. Knocking out *Pfs16* or *Pfs37* has shown a reduction in the number of oocysts generated in the mosquito midgut during the parasite invasion [15, 21]. Initially *Pfs16* was suspected to be required for optimal production of sexual stage parasites [22].

In addition to *Pfs16* and *Pfs37*, other genes such as *Pfs25* (PF3D7_1031000) and *Pfs28* (PF3D7_1030900) have also been studied in the context of parasite–vector interactions. *Pfs25* encodes a protein expressed on the surface of zygotes and ookinetes, playing a critical role in the development of oocysts in the mosquito midgut. Disruption of *Pfs25* results in significantly reduced transmission efficiency of the parasite [23]. Similarly, *Pfs28*, which is co-expressed with *Pfs25*, has been implicated in ookinete development and midgut invasion [24]. While *Pfs25* and *Pfs28* are known to be essential in the later stages of parasite development in the mosquito, we chose *Pfs16* and *Pfs37* for their earlier involvement in the interaction, particularly during gametocyte and ookinete stages, which are critical for initial midgut invasion. By focusing on *Pfs16* and *Pfs37*, our study aims to investigate earlier-stage interactions between *P. falciparum* and *An. gambiae*, complementing existing knowledge of transmission-blocking candidates such as *Pfs25* and *Pfs28*. This approach provides a more comprehensive understanding of the genes involved across different stages of the parasite’s lifecycle within the mosquito.

At the other end of the vector–parasite interaction equation, *Anopheles* midgut proteins *AnAPN1* and *HPX15* have been recognized for their importance in vector–parasite interactions and for their significant impact on the survival of the parasite. *HPX15* is an immune-related protein with pattern-recognition molecules, and previous studies indicate that it promotes malaria transmission [10]. Specifically, *HPX15* plays a role in the preservation of the functionality of stored sperm and long-term fertility in *An. gambiae* [25]. In *An. stephensi* mosquitoes, RNA interference-mediated silencing of midgut *AsHPX15* gene has drastically reduced the number of developing *P. berghei* oocysts [26]. Alanyl aminopeptidase N (AnAPN1) is a protein that can elicit transmission-blocking antibodies, which is believed to be highly conserved among *Anopheles* vectors [27], though not thoroughly investigated across Africa. The effectiveness of antibodies targeting *AnAPN1* against *P. falciparum* and *P. vivax* across distantly related *Anopheles* species is well studied [28].

While there is strong support of ancient vector-mediated selection on *Plasmodium* by continentally structured *Anopheles* species, questions remain about the potential for ongoing vector-mediated selection within a continental region. The goal of this study was to evaluate the potential for vector-mediated selection on parasite populations within African countries by examining the patterns of diversity in vector–parasite interacting genes. To better understand the subcontinental dynamics of vector-mediated selection, we aim to investigate the genetic diversity and selection signals in *P. falciparum* interaction genes, along with the vector species composition and subspecies variation in *An. gambiae* interaction genes. This study will specifically address the implications of *An. stephensi* invasion by providing insights into the nature of these interactions on a finer scale.

## Methods

All the command line-based programs were run on the operating system Rocky Linux 8.8 (Green Obsidian), Architecture: × 86–64. The rest of the steps were carried out on a Windows (version 11 Education, 64-bit operating system) PC.

### Data selection

We needed gene sequences from *P. falciparum* and *An. gambiae* from the malaria-endemic regions for this study. Therefore, Ag1000 and PF6K datasets shared by MalariaGen data-sharing network were used [29]. In this study, abiding to the Ag1000 terms of use, we did not use more than 10% of the genome data and we did not report any genome-wide statistics. When downloading genomes of vectors and parasites, sample sets were selected as separate populations, where at most 25 samples were collected from the same country in the same year. This number of samples (*n* = 25) was selected to hold the balance between representation of mosquitoes from a particular region and computational power required to perform the analysis. Another reason to select the same number (or close to 25) of samples for every population was to avoid the increment of number of haplotypes due to the large number of samples. The *P*. *falciparum* samples with high probability of multiple infections (*F*_ws_ ≤ 0.95) were removed from the dataset using the *F*_ws_ values calculated by the authors of the Pf6K dataset [29]. Abiding to above criteria, 418 *P. falciparum* genomes and 625 *An. gambiae* genomes were downloaded for 9 and 15 African countries, respectively. The European Nucleotide Archive (ENA) accession identifiers with country and year data are saved in Supplementary Table 1 and 2 CSV files.

### Quality control

The downloaded genome sequences were subjected to quality control using FastQC v0.12.1 [30] to assess the overall quality of the raw reads. Specifically, we evaluated key metrics including basic statistics (e.g., total sequence length, GC content), per-base sequence quality (to check for base-specific errors), per-base N content (to identify unknown bases), and sequence length distribution (to detect potential adapter contamination or other sequencing artifacts). Any anomalies observed, such as significant drops in sequence quality at specific positions or elevated N content, were flagged for further investigation.

To improve the quality of the dataset, we used Trimmomatic v0.39 [31] for trimming the low-quality regions. This included trimming sequences with Phred scores below 20, removing adapter sequences, and filtering out reads shorter than 36 bases post-trimming. The impact of these filtering steps was reassessed by generating a second round of FastQC reports to ensure that all quality anomalies were adequately addressed before proceeding with alignment.

### Sequence alignment to the reference genome.

The quality-filtered sequences were aligned to the *An. gambiae* and *P. falciparum* reference genomes, which were downloaded from the VectorBase (https://vectorbase.org/vectorbase/app) and PlasmoDB (https://plasmodb.org/plasmo/app) databases, respectively. Bowtie2 (version 2.5.1) [32] was used to perform the alignment on a Linux platform.

For the alignment, Bowtie2 was set to default parameters, which allow for end-to-end alignment. A maximum of two mismatches per read were permitted to balance sensitivity and accuracy. The resulting SAM files were converted to BAM format using SAMtools v1.10, followed by sorting and indexing to facilitate downstream variant calling and read depth analysis. Post-alignment quality checks, including alignment rates and mapping quality, were conducted to ensure that the majority of reads were correctly aligned to the reference genomes. Unmapped reads were excluded from further analysis.

### Variant calling and gene sequence handling

The variants were called from the aligned .bam files using the mpileup option in BCFtools (version 1.17) program [33]. Variants were normalized and filtered to get the highest quality variant call respective to the reference genomes. Only the biallelic variants were filtered out with Phred-scaled quality scores greater than 30, read depth greater than 10, and frequencies higher than 1%. The sequences of the interested g enomic regions were extracted from the VCF files using SAMtools program (version 1.18) and consensus option in BCFtools program. Extracted sequences were saved in FASTA format for the downstream analysis. VCF files of the mosquito genomes were phased using Segmented HAPlotype Estimation & Imputation Tool (shapeit2—version 2.r904) to address the ploidy level. The gene sequences were aligned using Clustal and Muscle programs [34, 35].

### Diversity statistics calculation

For all the genes studied here, for each population in both vectors and parasites, we calculated the Tajima’s *D* values, *F*_st_ values, nucleotide diversity, and haplotype diversity using the Pegas package (version 1.3) in R statistics (version 4.3.2) [36]. Additionally, genome-wide *F*_st_ and Tajima’s *D* values were calculated for comparisons. Values were recorded in tables for further analyses and visualized using the ggplot2 package in R statistics. Tajima’s *D* values were calculated according to the method described by Tajima in 1989 [37]. Tajima’s *D* values were recorded with the corresponding beta *P*-values for each population to facilitate selecting statistically significant signals of selection. Nucleotide diversity and haplotype diversities for each population pair were calculated as described in Nei in 1987 and Nei and Tajima in 1981, respectively [38, 39]. Pairwise *F*_st_ for each population pair was calculated using gene.dist() function in hierfstat (version 0.5.11) package in R statistics using the method described by Weir and Cockerham in 1984 [40]. Genome-wide Tajima’s *D* value and F_st_ values were calculated using VCFtools (version 0.1.16).

### Investigation of relationships between vector occurrence probabilities and parasite gene haplotype diversities

In this step we investigated the relationships between amino acid haplotype diversities of the genes important for interacting with the vectors in parasites and the probability of occurrence of prominent malaria vectors in Africa. We downloaded predicted vector occurrence probabilities (VOP) for both 2010 and 2017 from the Malaria Atlas Project, corresponding to the locations where parasite samples were collected [41, 42]. Correlation analyses were performed between the amino acid haplotype diversities of parasite genes and VOP using cor.test() function on R statistics platform. For the vector species that had a statistically significant relationship with the haplotype diversity of parasite genes, regression models were fitted using lm() function to examine the interaction between vector species occurrence probabilities on haplotype diversities of parasite genes. Furthermore, to reduce the uncertainty and noise inherent in VOP data that were used in linear regressions, the probabilities were converted to a binomial variable of presence or absence of the vector species. We employed three distinct cutoff values for occurrence probabilities (0.5, 0.75, and 0.95) to assess vector presence, as a definitive rationale for selecting a single threshold was not available. Regression models were fitted to predict the amino acid haplotype diversities of the parasite genes against binomial vector occurrence of significantly correlated vector species as the predictor variables. Results of all the regression models were tabulated in an Excel sheet (Supplementary data sheet 2—Combined sheet). In addition to the regression analyses, we categorized and visualized the haplotype diversities and Tajima’s *D* values of parasite populations on the basis of the presence or absence of different combinations of vector species significantly associated with parasite gene haplotype diversity (Supplementary Figs. 4 and 5). This approach was specifically designed to identify patterns in haplotype diversities across various vector combinations that were not captured by the linear regression models.

### * Anopheles stephensi* mosquito collection and P47Rec sequence extraction

*Anopheles stephensi* DNA generated from previous studies [6, 43] were used for the analyses described below. These source specimens were part of a September–November 2018 collection from northeastern and eastern Ethiopian cities Semera and Kebridehar as a part of our previously published studies as previously described [6, 43]. Briefly, mosquitoes (*n* = 7) were collected using Centers for Disease Control and Prevention light traps and pyrethrum spray collection in houses, and larvae and pupae were sampled using the WHO dipping approach. The mosquito specimens were collected and handled following ethical guidelines as previously described by Balkew et al. in 2020 [6], and a materials and data-sharing agreement was established between Baylor University and Jigjiga University. DNA was extracted from the dissected heads and thoraxes of the mosquitos using the Qiagen DNeasy kit. Once the DNA was extracted, the *P47Rec* ortholog in *An. stephensi* was amplified using two primer pairs. The first pair (forward—5′-TGGCAAATGACTAACGTGGA-3′, reverse—5′-GTGTTGCCAGTTCGCTGTAA-3′) amplified the second and third exons, while the second pair (forward—5-GTGAGCAGCTGTACGTTGGA-3′, reverse—5-AAAACGGAAGGCATGTCATAA-3′) amplified the fourth exon. Sequences were aligned using the MUSCLE program and a maximum likelihood tree was generated using the RAxML version 2.0 program [44].

## Results

### Population structure and polymorphism in *Plasmodium falciparum* genes

**Fig. 1:**
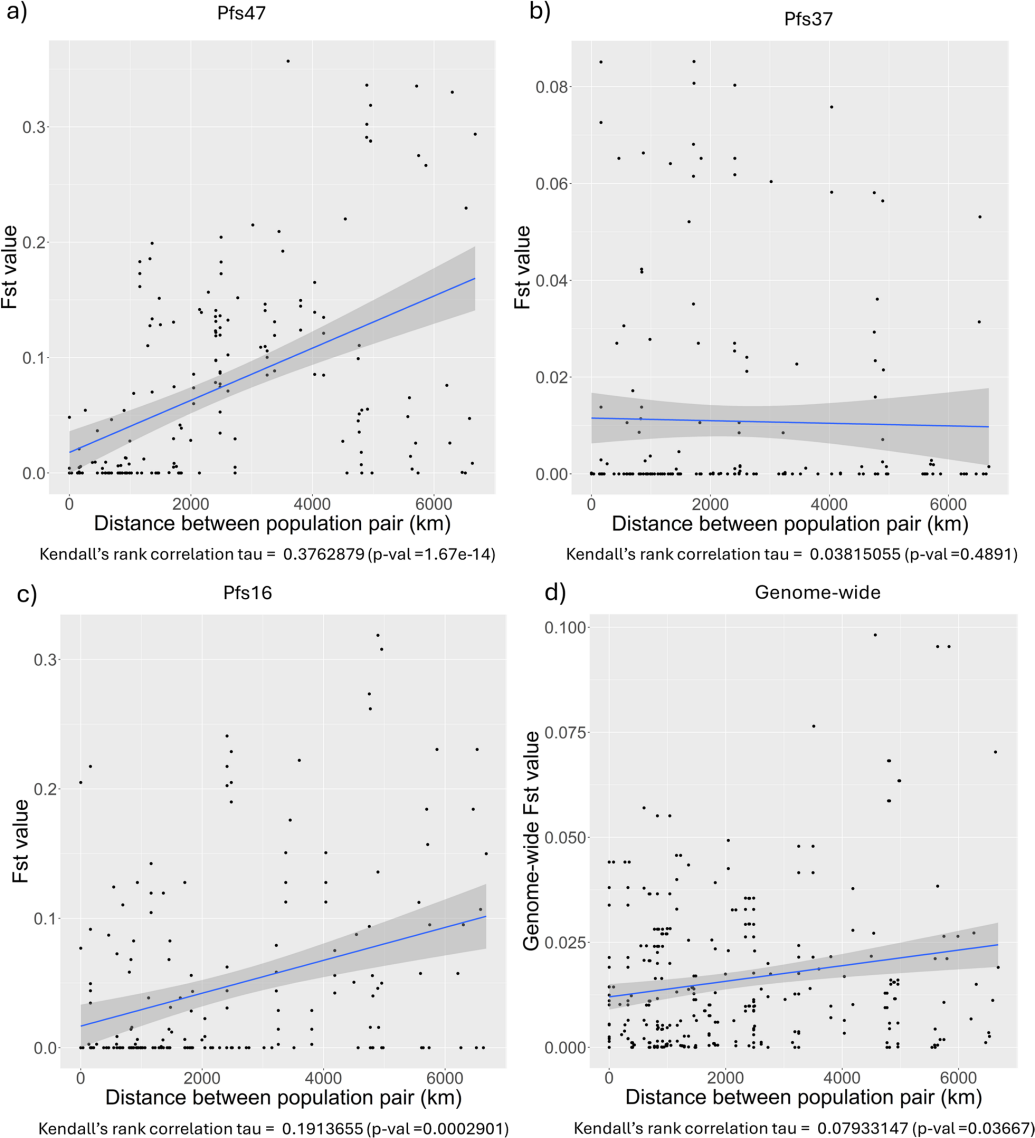
Analysis of genetic differentiation and geographic dispersion among populations of *P. falciparum* across Africa. Each panel depicts the correlation between pairwise fixation index (*F*_st_) values and geographic distances, measured in kilometers, between population pairs for different genomic regions. The *F*_st_ value measures the genetic differentiation between populations, with higher values indicating greater divergence. The blue line represents a regression line fitted to the data points, illustrating the relationship between geographic separation and genetic diversity. **a** Displays *F*_st_ values for the *Pfs47* gene, indicating a moderate positive correlation. **b** Shows *F*_st_ values for the *Pfs37* gene, with a weak and nonsignificant correlation. **c** Illustrates the correlation for the *Pfs16* gene, suggesting a mild positive correlation. **d** Presents the genome-wide correlation, indicating a slight but statistically significant correlation

To investigate the population structure and its association with geographic distribution of *P. falciparum* genes, we measured the pairwise *F*_st_ between each population pair and tested for correlation with geographic distance between populations (see Fig. 1). For *Pfs47*, pairwise *F*_st_ values ranged between 0 and 0.3569732. The highest values were observed in the Malawi population against other populations. Malawi was also relatively (not statistically) isolated from other Central African countries including the Democratic Republic of Congo and Cameroon, which were relatively (not statistically) isolated from West African populations (see Supplementary Fig. 1). In *Pfs47*, a statistically significant but weak correlation was observed between pairwise *F*_st_ values and the geographic distances among the *P. falciparum* populations (see Fig. 1).

In parallel to *Pfs47*, *Pfs16* also showed a statistically significant but weak correlation between pairwise *F*_st_ values and the geographic distances among the *P. falciparum* populations (Kendall’s rank correlation tau = 0.1913655, *P* = 0.0002901). Again, Malawi had the highest pairwise *F*_st_ values, but the isolation patterns were different between *Pfs47* and *Pfs16* (see Supplementary Fig. 1). Both *Pfs37* and genome-wide pairwise *F*_st_ values did not show statistically significant correlations with the distance between populations (Fig. 1).

**Fig. 2:**
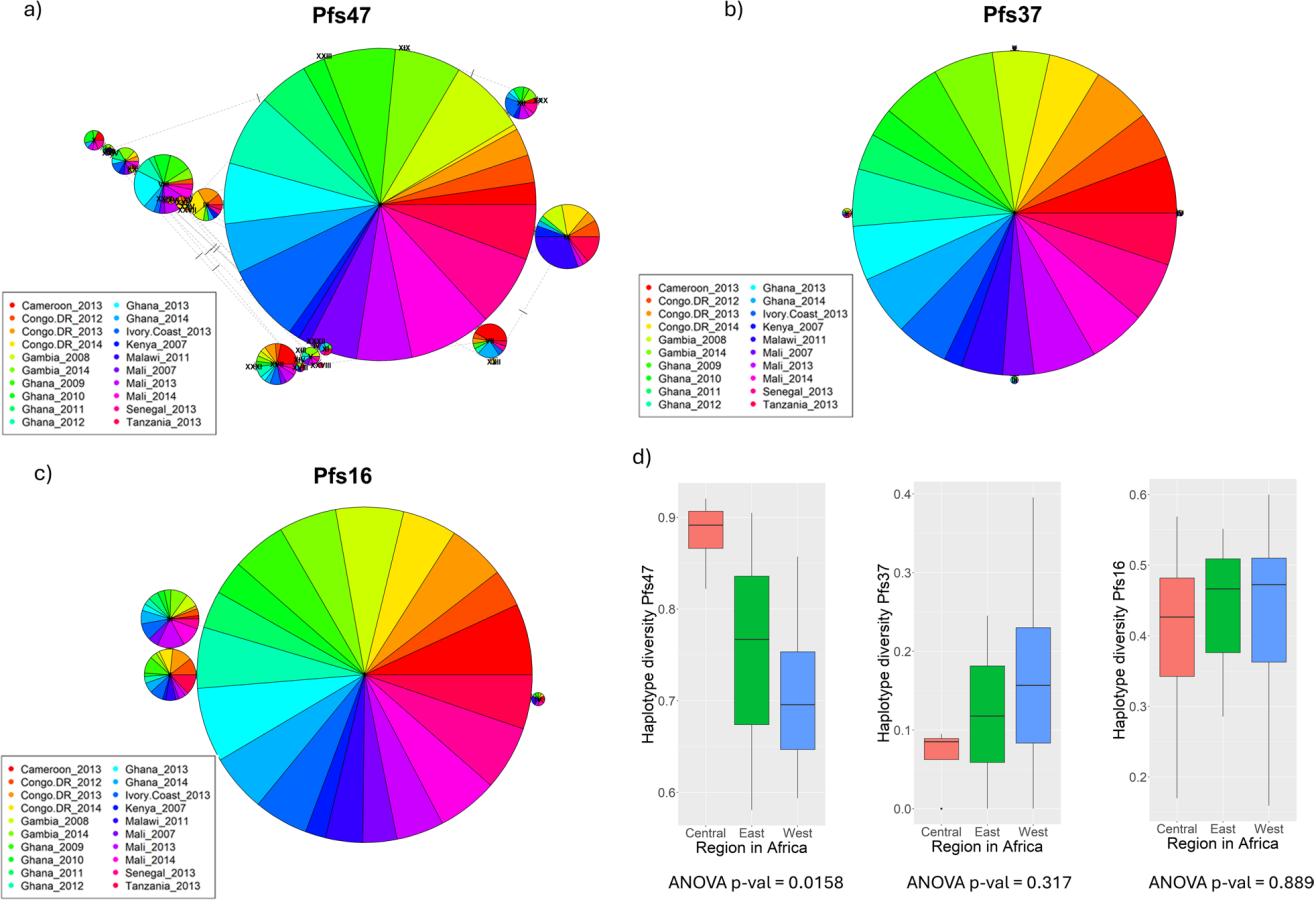
Haplotype diversity and distribution of *P. falciparum* genes *Pfs47*, *Pfs37*, and *Pfs16* across Africa (based on nucleotide sequences). These haplotype networks graphically represent how each haplotype has been represented by different populations of parasites. The size of the pie represents the number of individuals with the haplotype and the size of each pie segment reflects the relative frequency of parasites originating from different populations for each haplotype. **a** *Pfs47* had 32 haplotypes that are distributed among many populations. **b** *Pfs37* had a single prominent haplotype, suggesting very low genetic variability. **c** *Pfs16* also had a fewer number of haplotypes, suggesting less genetic variability compared with *Pfs47* but higher than *Pfs37*. **d** Regional haplotype diversity analysis—box plots represent the distribution of haplotype diversity levels for *Pfs47*, *Pfs37*, and *Pfs16* across three major regions: Central, East, and West Africa. Each box plot shows the median, quartiles, and potential outliers, providing a statistical summary of regional genetic diversity. ANOVA results below each plot indicate the statistical significance of differences in diversity across regions, with the *P*-values providing insights into regional variations in genetic diversity. (These calculations were based on nucleotide sequences and the calculations based on amino acid sequences are different from this.)

**Fig. 3:**
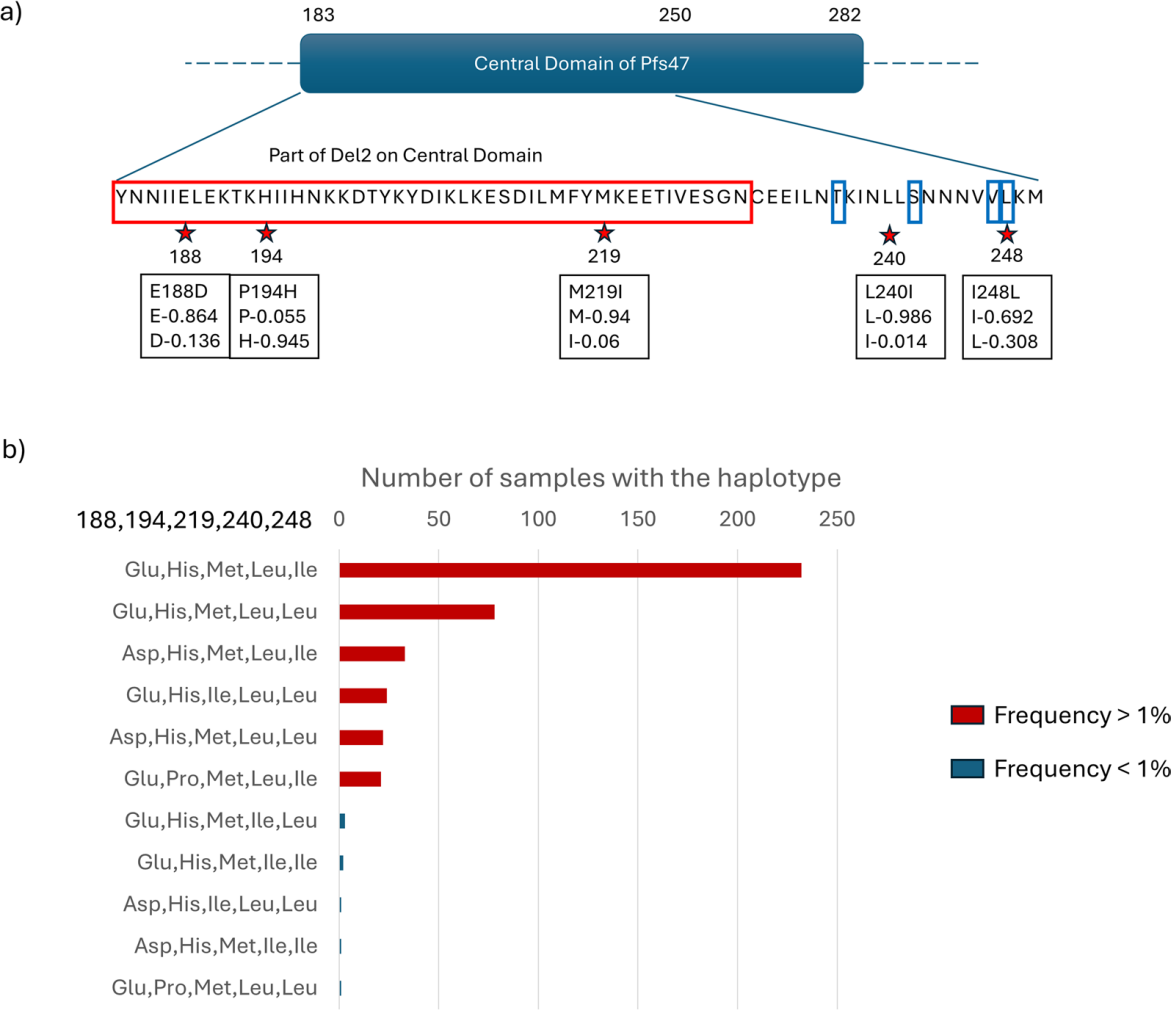
Analysis of non-synonymous mutations in the central domain of *Pfs47* and their frequencies. **a** The locations of the mutations on domain 2 of *Pfs47* protein. The red stars denote the polymorphic sites within the region (183rd aa to 250th aa) and red box denotes Del2 region, which is selected as antigen for TBV. The blue boxes show the previously identified mutations important for vector parasite interaction. Notation within the black boxes shows the mutation in the first line and frequency of each allele in second and third lines **b** This bar plot displays the distribution of haplotypes found within the 418 protein sequences of *Pfs47*’s central domain (D2), sampled from various populations across Africa. Haplotypes are categorized by the composition of their amino acid sequences at the polymorphic sites identified in panel (**a**). Bars are colored to distinguish haplotypes with a frequency greater than 1% (red) from those less frequent (blue), offering a visual summary of haplotype prevalence and diversity within the dataset

Among *P. falciparum* in the African countries we studied, the number of haplotypes for *Pfs47*, *Pfs37*, and *Pfs16* were 32, 5, and 4, respectively (Fig. 2). We observed three polymorphic sites within the Del2 region in the central domain of *Pfs47* (D2), which has been selected as a candidate antigen and which generates antibodies that block transmission (see Fig. 3) [11]. Secondly, we observed two amino acid polymorphisms between the two cysteines in *Pfs47*-D2, the region known to be important for mosquito infectivity (see Fig. 3). To understand the geographical distribution of haplotypes in parasite populations we generated haplotype networks for the three parasite genes. In all three haplotype networks, especially the *Pfs47* central domain (D2) haplotype network, there was roughly equal representation from all the populations, indicating the presence of each haplotype in many parts of the continent (see Fig. 2 and Supplementary Figs. 2, 5). In *Pfs47*, haplotype II had the highest number of samples and represented most of the populations in the dataset. To find out which parts of the African continent harbor the highest number of parasite gene haplotypes, we divided the continent into three regions (East, Central, and West) and measured the haplotype diversity. Haplotype diversity level of *Pfs47* was significantly higher in Central African countries [analysis of variance, ANOVA *F*_(2, 17)_ = 5.344 *P* = 0.0158, Tukey’s HSD test *F*-values of West-Central and East-Central were 0.0119208 and 0.1825349 respectively, see Fig. 2d]. Compared with *Pfs47*, both *Pfs37* and *Pfs16* had haplotype diversity evenly distributed across the continent (see Fig. 2d box plot haplotype diversity means for *Pfs37* and *Pfs16*). In *Pfs47* there were nine single nucleotide polymorphisms (SNPs) and five of them had frequencies higher than 10% in the sample set we analyzed. Out of the nine SNPs, eight were non-synonymous mutations. There was a single SNP in *Pfs16* coding sequence and an indel expanding from the 423rd base pair to the 428th base pair creating amino acid changes I85L, D140-, and K141-. All these variations had a frequency higher than 10%.

### Signals of selection in parasite genes

One of the goals of this study was to see whether the parasite interacting genes are evolving under positive selection in any of the African populations investigated here. Therefore, we calculated the Tajima’s *D* values for each parasite gene for each population and compared it with the average Tajima’s *D* values of the entire genome (−0.783259075) for the samples analyzed in this study. Tajima’s *D* values were calculated for each population and tabulated (see Supplementary data sheet 1). Among parasite populations, the average Tajima’s *D* over the entire genome varied between −1.320805 and −0.2383106. For each population, *Pfs47* and *Pfs37* did not show any statistically significant signals of non-neutral evolution (averaged Tajima’s *D* values −0.420956776 and −1.167216138 respectively, beta *P* > 0.05). However, *Pfs16* had significantly higher negative Tajima’s *D* values in all the individual populations (average Tajima’s *D* value = −2.960073673, beta *P* < 0.05), indicating that it is evolving non-neutrally in many parts of the continent. Since the *Pfs16* Tajima’s *D* values were significantly negative compared with the genome-wide Tajima’s *D* value, this could be an indication of positive selection at this locus.

### Population structure in *An. gambiae* genes

**Fig. 4:**
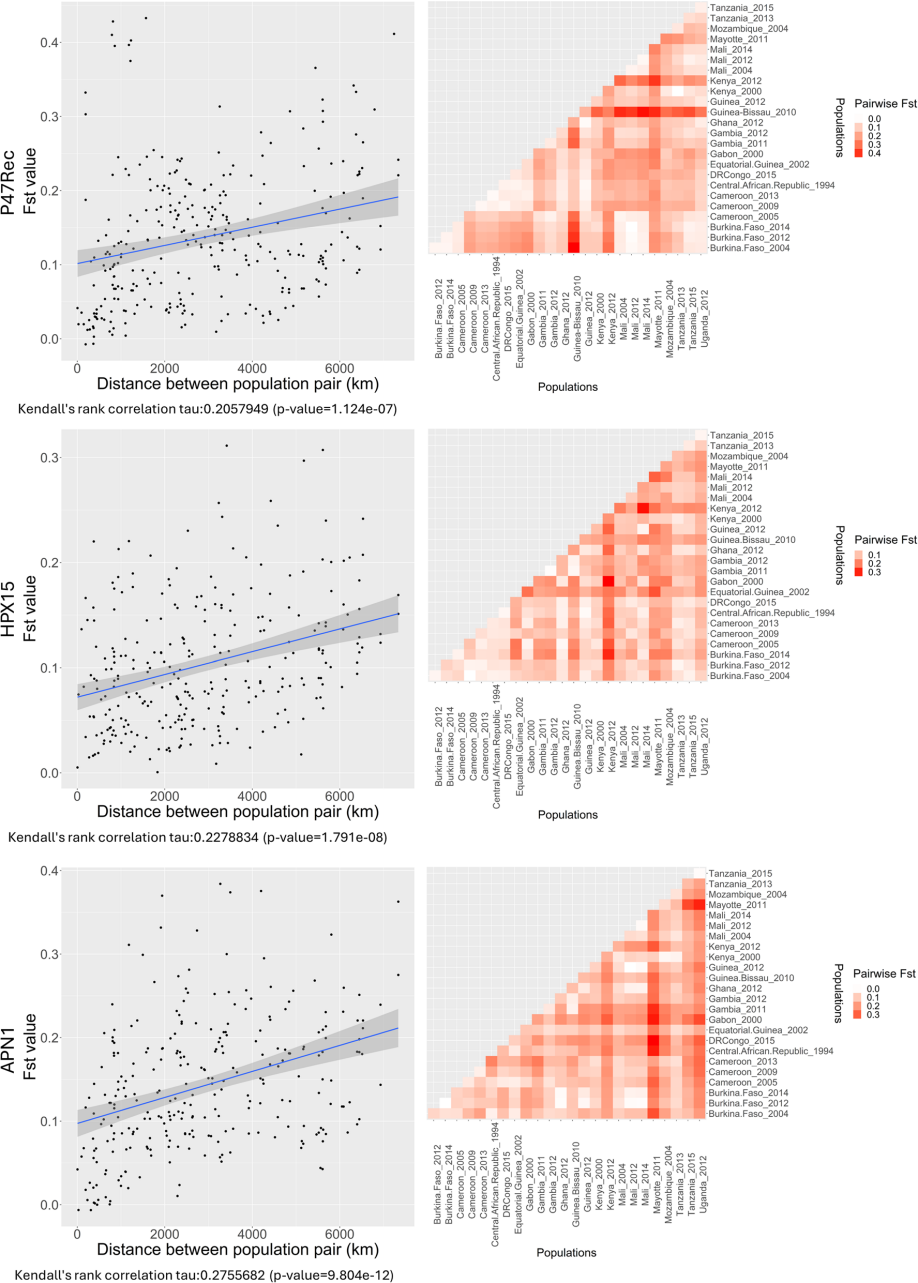
Genetic differentiation and the correlation between geographic distance and genetic differentiation for *An. gambiae* gene loci. Scatter plots: each scatter plot displays the correlation between pairwise *F*_st_ values and geographic distances among populations of *An. gambiae *s.s.. *F*_st_ values, measuring genetic differentiation, are plotted against the geographic distance in kilometers between each pair of sampled populations. The line represents a regression fit, indicating trends in genetic isolation by distance. Heat maps: These heat maps depict the pairwise *F*_st_ values between different populations of *An. gambiae* for the corresponding genes. Each cell in the matrix represents the *F*_st_ value between a pair of populations, with color intensity varying according to the scale of differentiation ranging from low (light) to high (dark). Populations are ordered and labeled on both axes, facilitating cross-reference between related populations

To investigate the potential influence of vector–parasite interactions on the population structure of *Plasmodium* parasites, we analyzed the population structure of genes (nucleotide sequences) coding for proteins known to be important for the survival of the parasite in *An. gambiae *s.s. in several African countries. We measured the pairwise *F*_st_ between each selected *An. gambiae* population pair and tested for correlation with the geographic distance between populations (see Fig. 4). The *F*_st_ values varied between 0 and 0.4327906, 0.3112532, and 0.3841258 for *P47Rec*, *HPX15*, and *APN1*, respectively. For the *P47Rec* gene, Guinea-Bissau and Kenya (2012) populations showed the highest level of isolation from other regions, while Central African populations had a trend of being differentiated from the other populations. In *HPX15*, the Kenya (2012) population was again the most isolated population compared with the rest of the populations, followed by Mayotte and Mozambique populations. However, in *APN1* Mayotte population had the highest level of isolation followed by the Southeast African populations in Uganda, Tanzania, and Mozambique. Kenya (2012) was relatively (not statistically) different from the rest of the populations. All three genes studied here showed statistically significant but weak correlations between the pairwise *F*_st_ values and geographic distance among populations (see Fig. 4).

The number of SNPs that cause changes in amino acids were tabulated for each exon of the three genes (see Table 1). No non-synonymous mutations were detected in the *P47Rec* with a frequency higher than 1%. However, 32 and 72 nonsynonymous mutations with frequency higher than 1% were detected in the *HPX15* and *APN1*, respectively. Given the amino acid variation in these two genes, we were interested in the phylogenetic relationship among the mosquitoes. Phylogenetic analysis revealed support for the presence of distinct clades within *An. gambiae* for *HPX15* (bootstrap values > 70) but not in APN1 (Supplementary Figs. 6 and 7).

**Table 1:** Number of non-synonymous mutations (NSMs) per exon in *An. gambiae* genes

Gene	Exon	# of NSMs	# of NSMs with freq. > 0.01
P47Rec	1	0	0
	2	2	0
	3	2	0
	4	5	0
	Total	9	0
HPX15	1	27	5
	2	44	17
	3	38	10
	Total	109	32
APN1	1	23	7
	2	18	10
	3	4	0
	4	25	3
	5	60	52
	Total	130	72

### Relationships between vector occurrence probabilities and parasite gene haplotype diversity

We conducted individual association analysis of the *P. falciparum* diversity statistics and occurrence probabilities of eight commonly occurring malaria vector species in Africa (*An. arabiensis*, *An. coluzzi*, *An. funestus*, *An. gambiae*, *An. melas*, *An. merus*, *An.0moucheti*, and *An. nili*). Only *Pfs47* and *Pfs16* amino acid haplotype diversities had significant associations (Kendall’s rank correlation *P* < 0.05) with predicted occurrence probabilities of *An. arabiensis*, *An. funestus*, and *An. moucheti* (see Table 2). Regression models were fitted to explain the amino acid haplotype diversities of *Pfs47* and *Pfs16* using the VOPs of the previously mentioned three vector species as continuous and binomial variables separately (Supplementary data sheet 2—Separate and Combined sheets). Models fitted with VOPs as continuous variables had significant overall *P*-values (< 0.05) and adjusted *R*-squared values greater than 0.48 but estimates for the VOPs were close to zero. In the regression models fitted with VOPs as binomial variables, irrespective of the cutoff value (to determine the presence or absence of a vector species from occurrence probabilities), *Pfs16* amino acid haplotype diversity was associated with *An. arabiensis* with a significant (< 0.05) *P*-value and an estimate close to zero while *Pfs47* was associated with *An. arabiensis* only under a cutoff value of 0.5. Apart from the linear regression analyses between parasite gene haplotype diversities and vector occurrence probabilities, we visualized the haplotype diversities observed under occurrence of different combinations of three vector species (*An. arabiensis*, *funestus*, and *moucheti*—occurrence was determined by multiple threshold values as previously mentioned) that were significantly associated with higher haplotype diversities (Supplementary Fig. 4). We observed in most cases, irrespective of the threshold level used to define the probable presence or absence of the vector species on the basis of their predicted occurrence probability, that the combination of *An. arabiensis* and *An. funestus* was associated with higher levels of amino acid haplotype diversity in *Pfs47* and *Pfs16* (Supplementary data sheet 2—ANOVA sheet).

**Table 2:** Observed significant Kendall’s rank correlation tau estimates and *P*-values for the amino acid haplotype diversities of parasite genes and predicted vector occurrence probabilities based on 2010 prediction model

	*An. arabiensis*	*An. funestus*	*An. moucheti*
Pfs47			
Tau estimate	−0.4217757	−0.3807641	0.434292
* P*-value	0.01082	0.02068	0.02132
Pfs16			
Tau estimate	0.6099269	0.5013643	−0.4863484
* P*-value	0.0002694	0.002899	0.01087
Pfs37			
NA	Nonsignificant	Nonsignificant	Nonsignificant

We investigated the relationship between Tajima’s *D* values of *Pfs16* and vector occurrences because it was the only gene that had signals of significant positive selection. We observed significant estimates for *An. funestus* VOP in both regression models fitted to explain *Pfs16* Tajima’s *D* using vector occurrence probabilities as continuous variables and binomial variables (see Supplementary data sheet 2). It is important to mention that *Pfs16* Tajima’s *D* values did not have a significant association with the region of Africa (Central, East, or West) as observed in the results of the one-way ANOVA table (*Pfs16* Tajima’s *D* values against region—see supplementary data sheet 2). However, *An. funestus* vector occurrence (as a binomial variable) had a significant relationship with region (analysis of variance *P* = 0.0493). In addition to the regression analyses mentioned above, we created a violin plot of the *Pfs47* haplotype diversity, *Pfs16* haplotype diversity, and *Pfs16* Tajima’s *D* values for different vector species combinations. In those plots, we observed that higher (i.e., less negative) Tajima’s *D* values and haplotype diversities at *Pfs16* were associated with the combinations that included *An. funestus* (Supplementary Fig. 5, Supplementary data sheet—ANOVA sheet).

### Comparative analysis of P47Rec in *An. gambiae* and *An. stephensi*

**Fig. 5:**
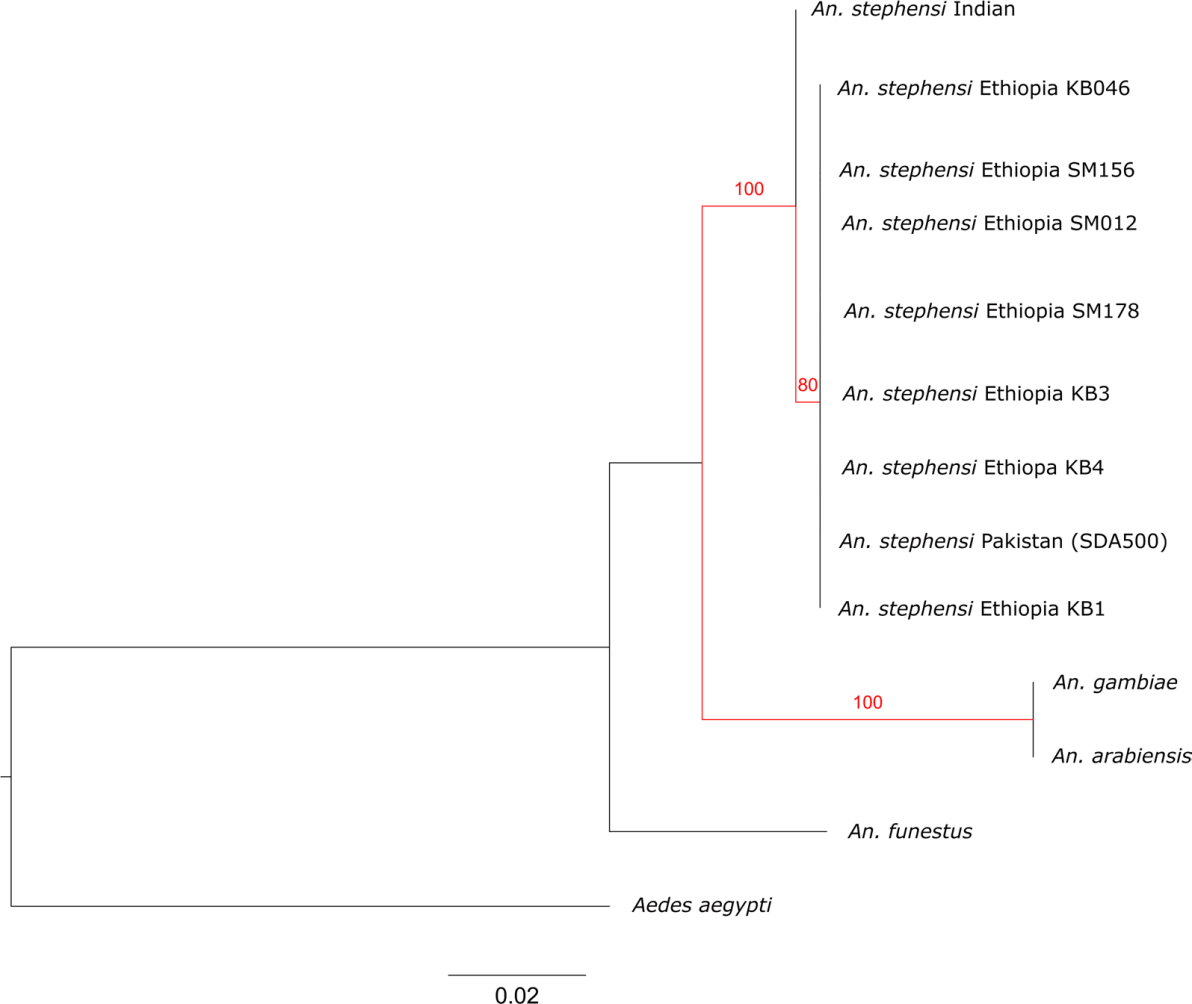
Maximum likelihood tree for the *P47Rec* orthologs of prominent malaria vectors in Africa with *P47Rec* ortholog of *An. stephensi* collected in Ethiopia (500 bootstraps). The significant branches with bootstrap values higher than 70 are highlighted in red. It is important to note that all the sequences from Ethiopia are closely related to SDA500 strain and significantly divergent from Indian reference sequence

In *An. gambiae* s.s., the *P47Rec* amino acid sequence was very well conserved within the sequences we observed in Ethiopia. With the invasion of *An. stephensi* into the Horn of Africa (HOA) we wanted to investigate the differences in amino acid sequence of *P47Rec* ortholog in *An. stephensi* that could have an impact on compatibility between invasive vector and existing *Plasmodium* populations in the HOA. There are 28 amino acid changes among all the prominent malaria vectors in Africa (*An. funestus, An. melas, An. quadriannulatus, An. arabiensis, An. merus*, and *An. gambiae*) in *P47Rec* orthologs reported by Molina-cruz et al. in 2020 [16]. There were 18 amino acid changes (listed in Table 3) observed in *P47Rec* amino acid sequences between the *An. stephensi* collected in Ethiopia and the *An. gambiae* reference sequence compared with 25 observed between *An. gambiae* and *An. funestus*. Out of the 18 observed between *An. gambiae* and *An. stephensi*, 13 amino acid differences overlapped with the changes observed among all prominent African vectors mentioned above. There were five amino acid changes that are unique to *An. stephensi* (Table 3). No differentiation was observed between *An. stephensi* from Kebridehar and Semera. Further, we performed phylogenetic analysis of the coding sequence of the *P47Rec* gene (Fig. 5) including *An. gambiae *sl, *An. funestus*, *An. arabiensis*, and *An. stephensi* (sequences from Indian strain, Pakistani strain, and Ethiopia). The results indicated that the haplotype of the P47Rec ortholog in *An. stephensi* from Ethiopia is closer to that of *An. stephensi* from Pakistan (SDA500) as compared with the Indian one (separation was supported by bootstrap value = 80).

**Table 3:** Amino acid differences between *An. gambiae* and *An. stephensi* coding sequences of P47Rec

Exon	Amino acid change (from *gambiae* to *stephensi*)
Exon 2	G23A ~
	I34V ~
	Q53H*
	T67S*
Exon 3	S81G ~
	N90G ~
	I105V ~
	I119V*
	G131N ~
Exon 4	V165I ~
	N169H ~
	G173S ~
	T181A ~
	S195T ~
	T217V ~
	Q220N ~
	S242T*
	A252T*

The amino acid changes marked with * are changes unique to *An. gambiae* and *An. stephensi* collected in Ethiopia, and amino acid changes marked with ~ are observed among the other prominent malaria vectors (*An. funestus, An. melas* *quadriannulatus, An. arabiensis, An. merus*) and *An. gambiae*

## Discussion

### Limited evidence of vector-mediated selective pressure on *P. falciparum* populations through P47 system within Africa

The P47 system was the best starting point to investigate vector-mediated selective pressure on parasite variation on a subcontinental scale because of the well-established molecular interaction of the *P. falciparum* *Pfs47* and *An. gambiae* *P47Rec* proteins and their role in parasite invasion of the mosquito midgut. In this study, we observed 32 *Pfs47* haplotypes overall but no correlation between haplotype distribution and geography. In addition, no evidence of selection was detected according to the Tajima’s *D* values observed in the parasite populations studied here. Furthermore, the *P47Rec* amino acid sequence is highly conserved among *An. gambiae* populations, indicating the absence of vector subpopulation variation necessary to drive selection by *An. gambiae* alone. Even though we see *Pfs47* haplotype diversity within each population, the pattern of diversity in *Pfs47* suggests only neutral processes at play. This does not necessarily contradict previous studies that show evidence of differing *P. falciparum* haplotype compatibility across continentally structured *Anopheles* species [9]. The study by Molina-Cruz et al. identified a broad diversity of *Pfs47* haplotypes across East, Central, and West Africa, revealing substantial geographic variation in haplotype frequencies [17]. However, in our study, we intentionally limited the sample size, as one primary objective was to assess the relationship between *P. falciparum* gene haplotype diversity and VOP. Including disparate sample sizes from various populations may have inflated haplotype diversity estimates, particularly in regions with higher sampling densities. Therefore, we maintained a balanced sampling strategy to minimize such biases. While there are divergent *Anopheles* species that exist within Africa, the portions of the *P47Rec* that drive compatibility may be conserved in African *Anopheles,* leading to less restrictions on transmission specificity and the maintenance of diversity in *Pfs47*. Further functional analysis of *P47Rec* is needed to evaluate the precise genic regions at play in compatibility.

Interestingly, we also detected a new mutation (M219I) not yet reported in the central domain of the *Pfs47* Del region, which has been chosen to use as target antigen for the development of a malaria TBV [11]. Both methionine and isoleucine are uncharged, hydrophobic amino acids with non-polar side chains. However, the substitution of methionine with isoleucine may still affect protein function and structure due to subtle differences in their physicochemical properties, such as side chain size and flexibility. Methionine contains sulfur, while isoleucine is a branched-chain amino acid, which could influence protein folding, stability, or interactions with other molecules. Further investigation is needed to fully understand the potential impact of this substitution. Thus, these findings may have implications for the efficacy of vaccines, given that the native vectors can transmit this strain with these variants.

### Diversity in *An. gambiae* genes HPX15 and APN1

In contrast to *P47Rec*, both *HPX15* and *APN1* were highly diverse at the amino acid level. Phylogenetic analysis supported multiple distinct groups for *HPX15* (bootstrap values > 70) but not for *APN1* (bootstrap values < 70). The observed subspecies differentiation in *HPX15* indicates the potential for this gene to serve as a driver of selection on its matching *P. falciparum* gene in the parasite. Further investigation of this locus coupled with the identification of its corresponding *P. falciparum* surface proteins will elucidate whether vector-population-mediated selective pressure is occurring. We anticipate detecting signatures of balancing selection in the *HPX15* ligand(s) protein within *P. falciparum* populations across Africa due to the subpopulation variation observed at this locus on *An. gambiae*.

### Potential signals in *P. falciparum* Pfs16

While the study of the P47 system revealed limited evidence of ongoing vector-population-mediated selection on the *Pfs47* within Africa, differing patterns of diversity were observed at *Pfs16*. Fewer amino acid haplotypes were observed at this location and no geographic structure. Furthermore, a signal of positive selection or population expansion was observed in each study population at *Pfs16*. After comparing the *Pfs16* Tajima’s *D* values with genome-wide values, we could rule out the possibility of observing negative Tajima’s *D* values due to population expansion. This indicates that selection has occurred more recently at this locus within Central Africa. We also observed two important mutations, a single nonsynonymous mutation and a nine nucleotide (three amino acid) indel. While the receptor for *Pfs16* in vectors has not been identified yet, these findings support further investigation of this gene and potential receptors in the vector.

### Association analysis supports vector-mediated selection

Nucleotide and haplotype diversity in *P. falciparum* genes crucial for vector–parasite interactions could be significantly influenced by the composition of vector species in malaria-endemic regions. However, we were unable to obtain vector composition data for all the locations where the *P. falciparum* sequences and samples used in this study originated. To test the above hypothesis, we downloaded the predicted vector occurrence probability values of the prominent malaria vectors for the locations of parasite samples from the Malaria Atlas Project (MAP) and investigated the relationships between haplotype diversities and Tajima’s *D* values of the genes important for the *P. falciparum* interaction with vectors in Africa [41, 42]. We observed a statistically significant correlation between amino acid haplotype diversities of parasite genes *Pfs47* and *Pfs16*, and predicted occurrence probabilities of the vector species *An. arabiensis*, *An. funestus*, and *An. moucheti* individually. In addition to the individual associations, the above observation was confirmed by the association between higher haplotype diversities at *Pfs16* and combined occurrence of *An. arabiensis* and *An. funestus* compared with the occurrence of a single vector. These signals could be an indication of vector-mediated selection on parasite populations. However, we cannot rule out the impact of geography-correlated variables (e.g., transmission intensity, ancient genetic variation clines) on diversity due to the significant *F*-values observed in one-way ANOVA between amino acid haplotype diversity and the region of the African continent (Central, East, and West).

To investigate the impact of VOP on selection more directly, we examined the relationship between Tajima’s *D* values of *Pfs16* (*Pfs16* was the only gene with significant Tajima’s *D* values) and VOP of mosquito species that had a statistically significant correlation with parasite gene haplotype diversities individually. We found an association between *An. funestus* and *Pfs16* Tajima’s *D* values, such that the presence of *An. funestus* was associated with a lower signal of positive selection. *Anopheles funestus* is genetically distinct from *An. arabiensis* and *An. moucheti*, both members of the *An. gambiae* complex. This genetic diversity may have driven *P. falciparum* to develop a broader array of *Pfs16* haplotypes, enhancing its ability to be transmitted by both the *An. gambiae* complex and *An. funestus*. Consequently, the presence of multiple haplotypes could lead to a lower signal of positive selection in *P. falciparum* *Pfs16*. A limitation in this analysis is the availability of data on the lower end of *An. funestus* occurrence probability distribution. We have a single data point that is coming from Kilifi in Kenya (the only data point from Kenya). According to both the Malaria Atlas Project prediction of 2010 [41] and 2017 [42], Kenya has a low occurrence probability of *An. funestus*. It is also important to mention that the VOPs in the Malaria Atlas Project were predicted values based on geographical and environmental factors, which could add more ambiguity/noise to the estimate values of correlation analysis and regression analysis. Ultimately, these results indicate that there could be an influence from the vector species composition on the selection of the parasite genes important for the interaction with vectors.

### Implications for *An. stephensi* invasion in Africa

Given that *Pfs47* in African *P. falciparum* populations exhibited signals of neutral evolution in relation to the current sympatric vector populations (*An. gambiae*, *An. funestus*, etc.), we aimed to investigate how the evolution of *Pfs47* might be influenced by the introduction of *An. stephensi*. As an initial step, we examined the *P47Rec* ortholog in *An. stephensi*. Since the *P47Rec* coding sequence in *An. gambiae* in Africa was fully conserved, we wanted to investigate the number of amino acid changes in *P47Rec* ortholog in invasive *An. stephensi*. We compared the amino acid sequence of the *P47Rec* ortholog in *An. stephensi* from Ethiopia with the *An.gambiae* sequence and found 18 amino acid differences. These findings combined with phylogenetic analysis indicating differentiation between *An. stephensi* and the African species (*An. gambiae* bootstrap value = 100 and *An. funestus* bootstrap value = 100) support the potential for new *P. falciparum* haplotype compatibilities in Africa with the arrival, spread, and establishment of the invasive *An. stephensi*. In addition, the phylogenetic analysis revealed a close relationship between *P47Rec* in the invasive *An. stephensi* and the SDA500 *An. stephensi* strain (bootstrap = 100). The SDA500 strain is known to be highly susceptible to both I248L haplotypes in *Pfs47* in *P. falciparum* [18]. If the *Pf47Rec* was the gene that underwent artificial selection leading to higher susceptibility, it is possible the same patterns of susceptibility would be observed in *An. stephensi* with the similar *P47Rec* sequence. Therefore, the presence of similar *P47Rec* sequences (leading to high susceptibility) in the invasive *An. stephensi* may facilitate the gradual emergence of more *Pfs47* haplotypes in Africa. However, other genes may also influence the susceptibility of the SDA500 strain. To accurately determine the characteristics of *An. stephensi*—*Plasmodium* compatibility in Ethiopia, experimental infections are necessary to validate these hypotheses.

### Future directions

In this study we focused on several genes known to be important for vector–parasite interactions of malaria and their role in shaping the population structure of *P. falciparum* parasites through selective forces exerted from vector populations. There could be many other genomic factors that can influence the vector–parasite interactions and studies should include investigation of additional genes such as *Pfs25*, *Pfs28*, and circumsporozoite and TRAP-related protein (CTRP). This study did not include samples from all the malaria endemic countries in Africa. In addition, the samples were collected in multiple years that expanded over two decades. In a future study we expect to broaden our list of genes used in the analyses and to include samples from other malaria endemic countries in Africa.

## Conclusions

This study provides preliminary insight into the potential for vector subspecies level and multiple vector species selective pressure impacting *Plasmodium*–*Anopheles* compatibility within Africa. Notably, these findings support the notion that compatibility is complex and in addition functional, and population genetic investigations are needed. Our objective was to explore genetic diversity and related metrics as an initial step. It is crucial to recognize that definitive conclusions about compatibility cannot be drawn without experimental infection data. Furthermore, this study provides the first analysis to explore how occurrence of multiple vectors and invasive *An. stephensi* could change parasite diversity in multiple African countries. Finally, the current structure of diversity revealed that these transmission-relevant loci have major implications for the design and efficacy of vaccines and antimalarial treatments in *An. stephensi*-invaded regions.

## Supplementary Information

**Supplementary Material 1**: Figure 1: Pairwise Fst values for Pfs47, Pfs37 and Pfs16. Values indicated by color (white = low, red = high, grey = NaN).

**Supplementary Material 2**: Figure 2: The number of samples for each haplotype observed in *P. falciparum* populations for Pfs47, Pfs37 and Pfs16 genes. Bars are colored by the populations.

**Supplementary Material 3**: Figure 3: Haplotype network of Pfs47 central domain (D2).

**Supplementary Material 4**: Figure 4: Haplotype diversities of parasite genes (Pfs47 and Pfs16) against presence or absence (determined by VOP cutoff values 0.5, 0.75, and 0.95 separately) of combinations of only the vector species significantly associated with haplotype diversities. Some comparison categories were dropped due to lack of data points. Note: There are many other vector species that could be present in the same location and that were not considered in this analysis.

**Supplementary Material 5**: Figure 5: Tajima’s *D* values of Pfs16 against presence or absence (determined by VOP cutoff values 0.5, 0.75, and 0.95 separately) of combinations of vector species significantly associated with haplotype diversities. Some comparison categories were dropped due to lack of data points. Note: There are many other vector species that could be present in the same location and that were not considered in this analysis.

**Supplementary Figure 6.**

**Supplementary Figure 7.**

**Supplementary Table 1.**

**Supplementary Table 2.**

**Supplementary Data Sheet 1.**

**Supplementary Data Sheet 2.**

**Supplementary Material P47Rec ortholog Coding Sequences.**

## Data Availability

No datasets were generated or analyzed during the current study.
