# Supplementary figures and images for: Multi-locus investigation of Anopheles-mediated selective pressure on Plasmodium falciparum in Africa
Source: Parasit Vectors. 2024 Dec 23;17:530. doi: 10.1186/s13071-024-06604-y (PMC11665118; doi:10.1186/s13071-024-06604-y)

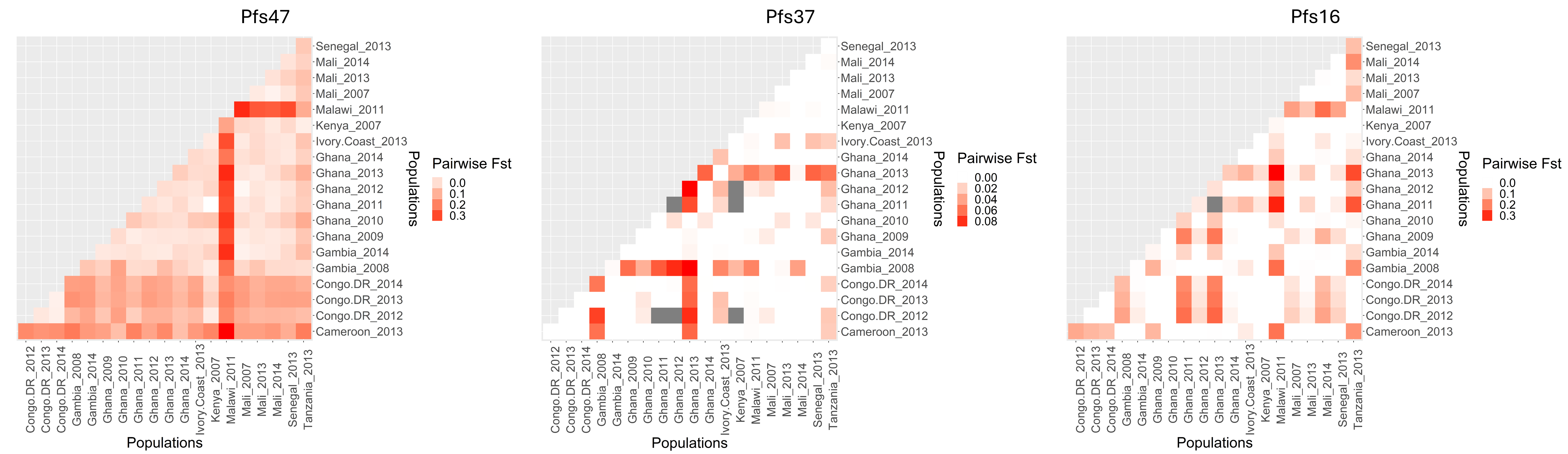

Supplement: Supplementary file 1 — Supplementary Material 1: Figure 1: Pairwise Fst values for Pfs47, Pfs37 and Pfs16. Values indicated by color (white = low, red = high, grey = NaN). [file 13071_2024_6604_MOESM1_ESM.png]

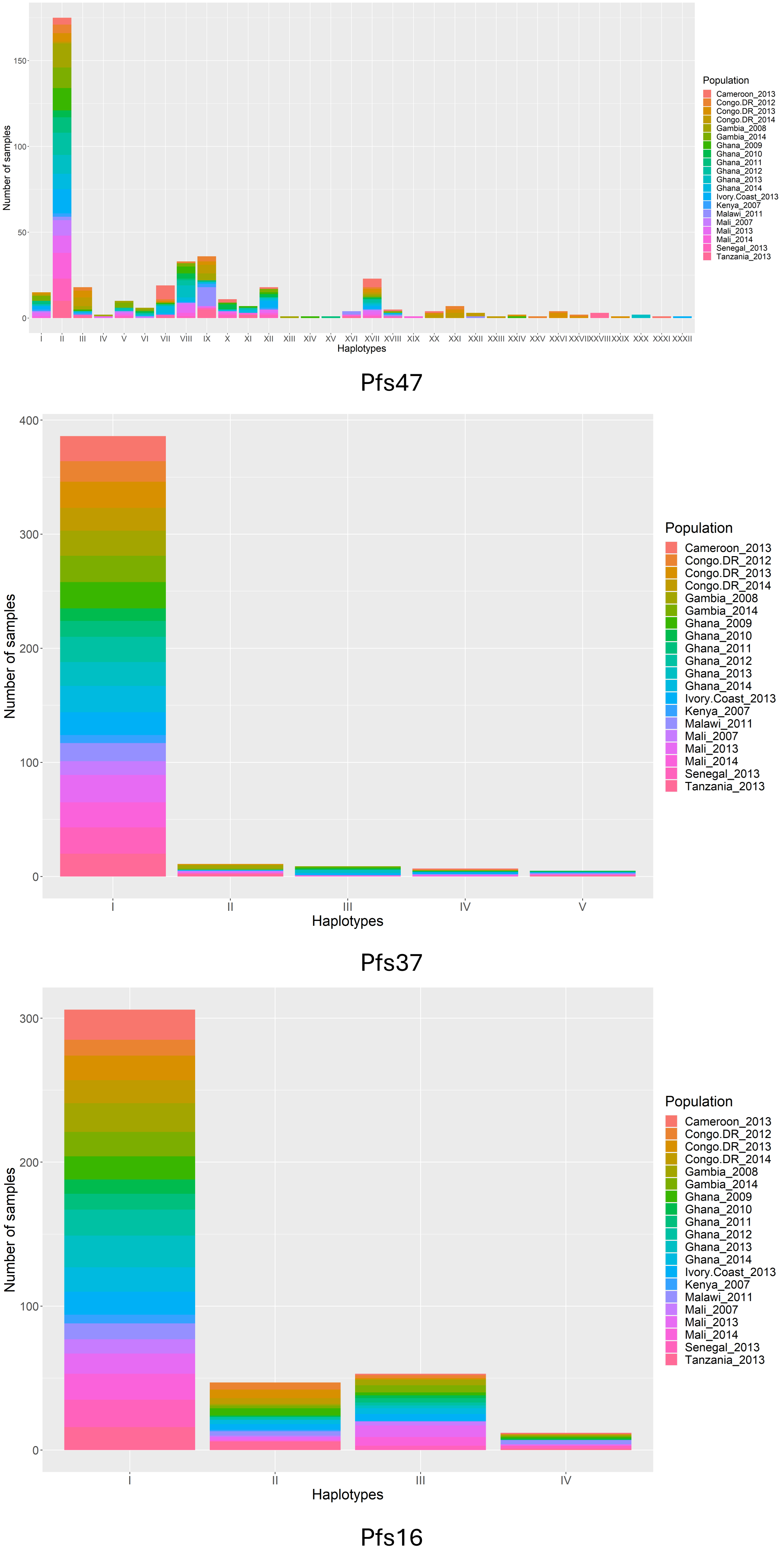

Supplement: Supplementary file 2 — Supplementary Material 2: Figure 2: The number of samples for each haplotype observed in P. falciparum populations for Pfs47, Pfs37 and Pfs16 genes. Bars are colored by the populations. [file 13071_2024_6604_MOESM2_ESM.png]

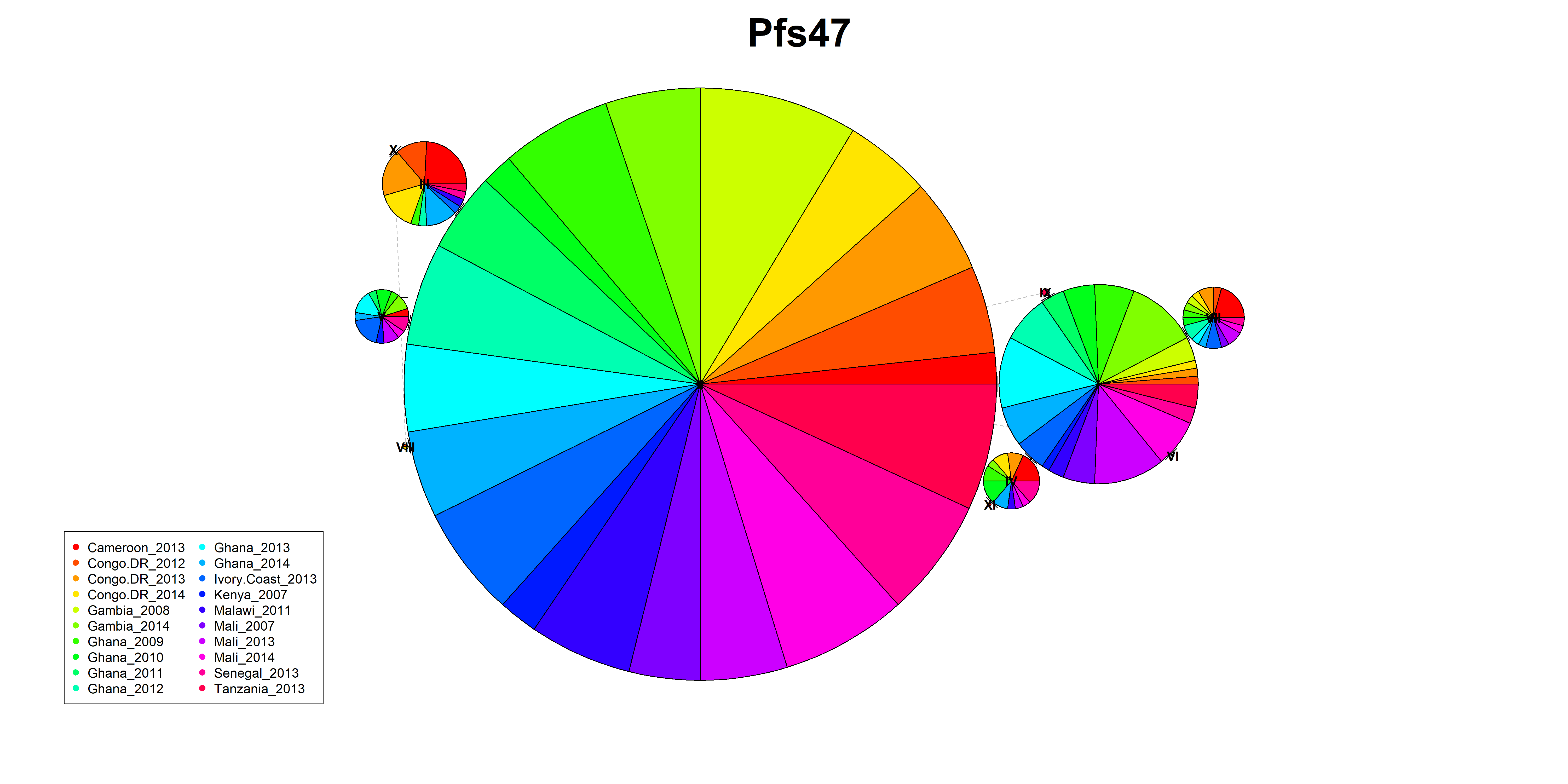

Supplement: Supplementary file 3 — Supplementary Material 3: Figure 3: Haplotype network of Pfs47 central domain (D2). [file 13071_2024_6604_MOESM3_ESM.png]

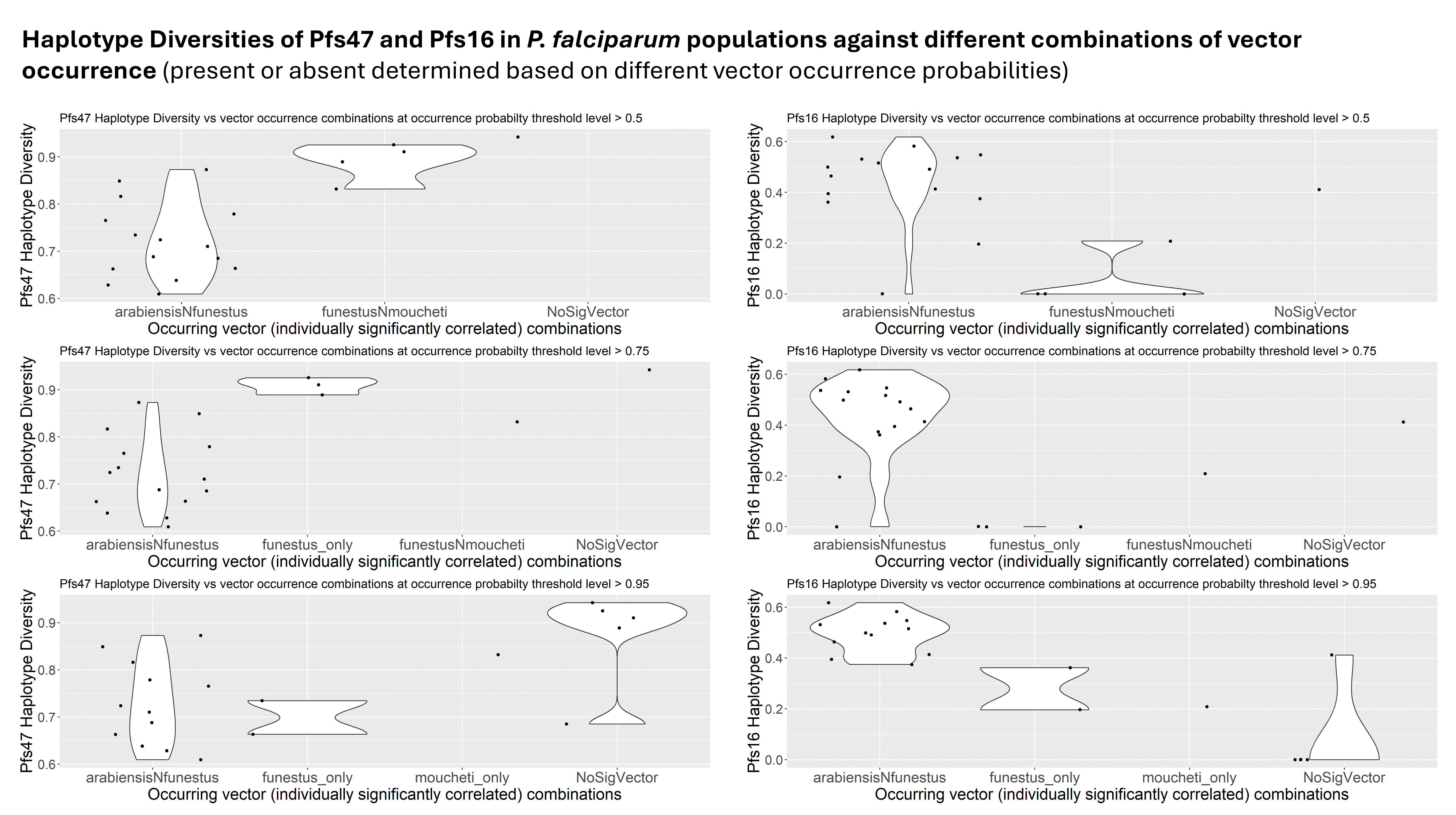

Supplement: Supplementary file 4 — Supplementary Material 4: Figure 4: Haplotype diversities of parasite genes (Pfs47 and Pfs16) against presence or absence (determined by VOP cutoff values 0.5, 0.75, and 0.95 separately) of combinations of only the vector species significantly associated with haplotype diversities. Some comparison categories were dropped due to lack of data points. Note: There are many other vector species that could be present in the same location and that were not considered in this analysis. [file 13071_2024_6604_MOESM4_ESM.png]

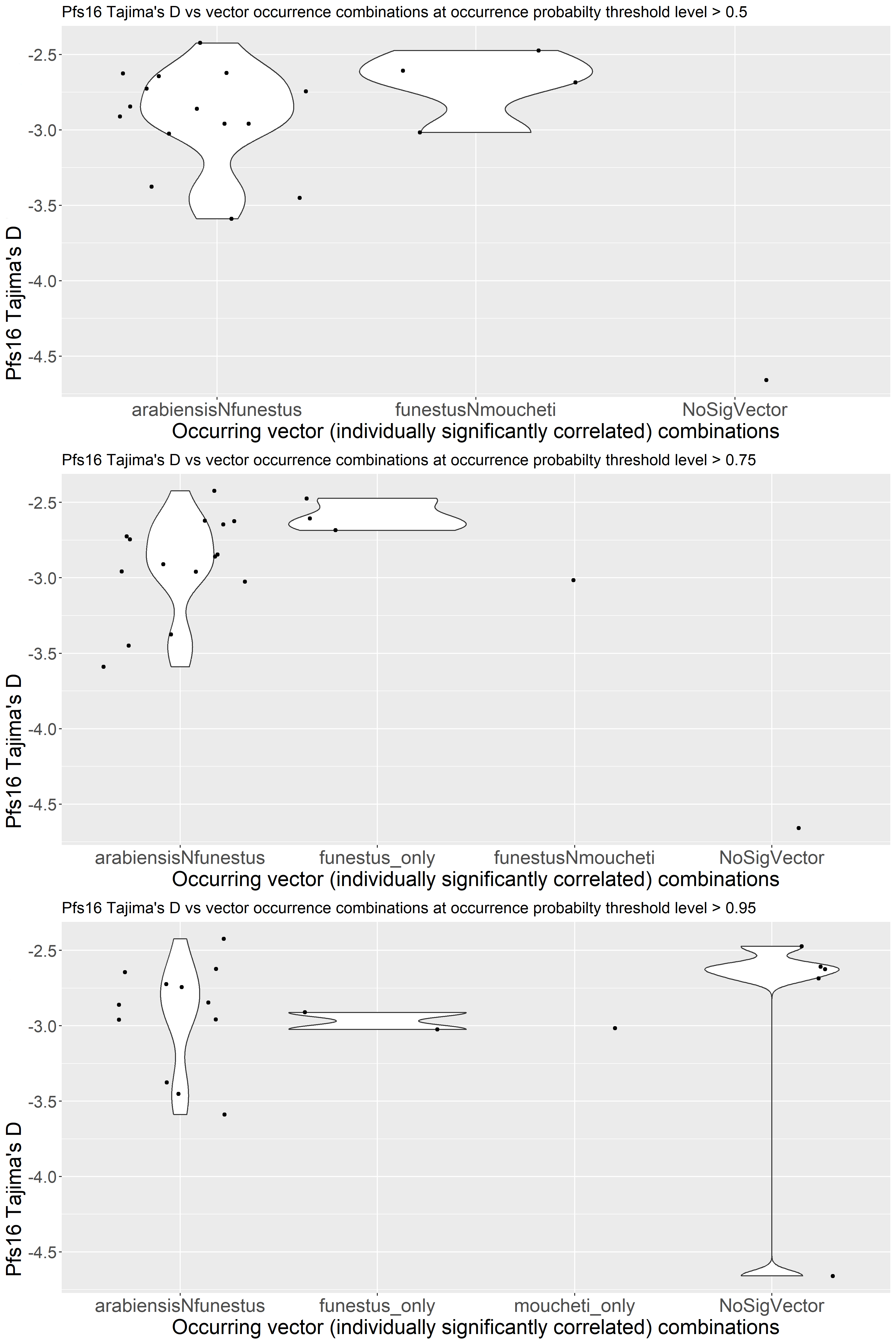

Supplement: Supplementary file 5 — Supplementary Material 5: Figure 5: Tajima’s D values of Pfs16 against presence or absence (determined by VOP cutoff values 0.5, 0.75, and 0.95 separately) of combinations of vector species significantly associated with haplotype diversities. Some comparison categories were dropped due to lack of data points. Note: There are many other vector species that could be present in the same location and that were not considered in this analysis. [file 13071_2024_6604_MOESM5_ESM.png]
