# Supplementary material for: Multi-locus investigation of Anopheles-mediated selective pressure on Plasmodium falciparum in Africa
Source: Parasit Vectors. 2024 Dec 23;17:530. doi: 10.1186/s13071-024-06604-y (PMC11665118; doi:10.1186/s13071-024-06604-y)
Supplement: Supplementary file 6 — Supplementary Figure 6. [file 13071_2024_6604_MOESM6_ESM.pdf]

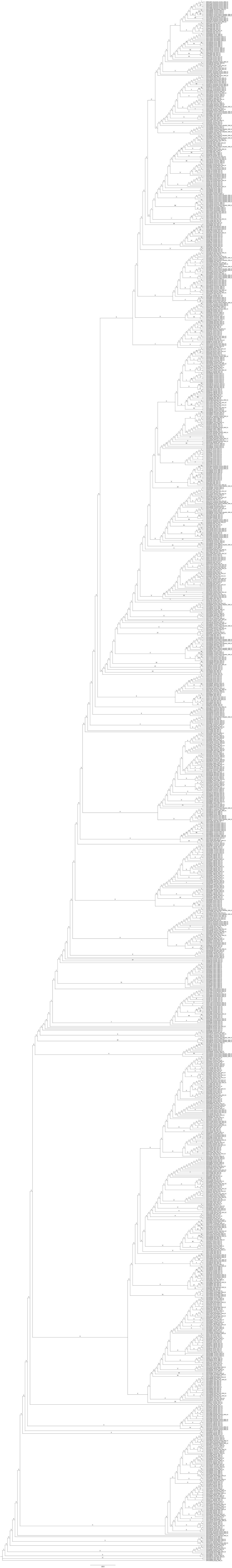
10.0
