# Supplementary material for: Multi-locus investigation of Anopheles-mediated selective pressure on Plasmodium falciparum in Africa
Source: Parasit Vectors. 2024 Dec 23;17:530. doi: 10.1186/s13071-024-06604-y (PMC11665118; doi:10.1186/s13071-024-06604-y)
Supplement: Supplementary file 7 — Supplementary Figure 7. [file 13071_2024_6604_MOESM7_ESM.pdf]

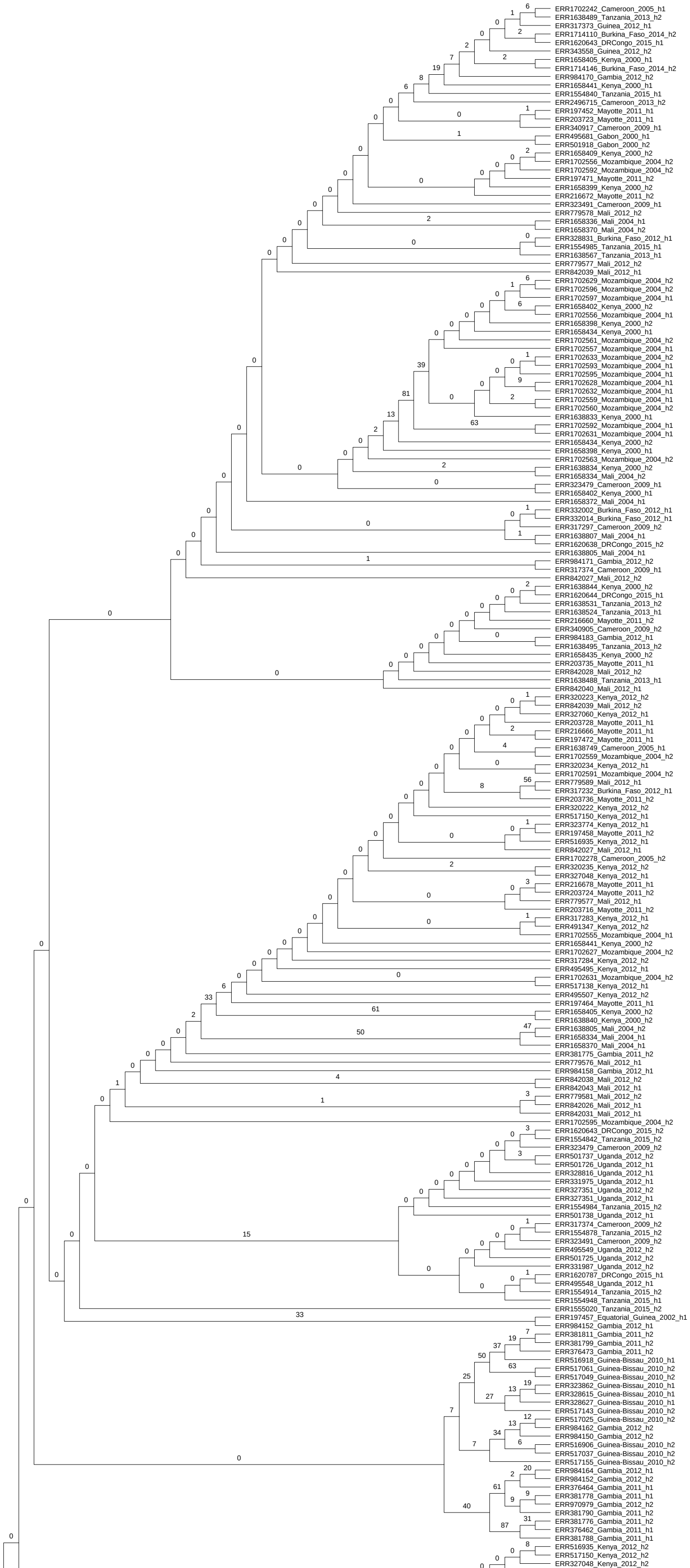
ERR1702242_Cameroon_2005_h1
ERR1638489_Tanzania_2013_h2
ERR317373_Guinea_2012_h1
ERR1714110_Burkina_Faso_2014_h2
ERR1620643_DRCongo_2015_h1
ERR343558_Guinea_2012_h2
ERR1658405_Kenya_2000_h1
ERR1714146_Burkina_Faso_2014_h2
ERR984170_Gambia_2012_h2
ERR1658441_Kenya_2000_h1
ERR1554840_Tanzania_2015_h1
ERR2496715_Cameroon_2013_h2
ERR197452_Mayotte_2011_h1
ERR203723_Mayotte_2011_h1
ERR340917_Cameroon_2009_h1
ERR495681_Gabon_2000_h1
ERR501918_Gabon_2000_h2
ERR1658409_Kenya_2000_h2
ERR1702556_Mozambique_2004_h2
ERR1702592_Mozambique_2004_h2
ERR197471_Mayotte_2011_h2
ERR1658399_Kenya_2000_h2
ERR216672_Mayotte_2011_h2
ERR323491_Cameroon_2009_h1
ERR779578_Mali_2012_h2
ERR1658336_Mali_2004_h1
ERR1658370_Mali_2004_h2
ERR328831_Burkina_Faso_2012_h1
ERR1554985_Tanzania_2015_h1
ERR1638567_Tanzania_2013_h1
ERR779577_Mali_2012_h2
ERR842039_Mali_2012_h1
ERR1702629_Mozambique_2004_h2
ERR1702596_Mozambique_2004_h2
ERR1702597_Mozambique_2004_h1
ERR1658402_Kenya_2000_h2
ERR1702556_Mozambique_2004_h1
ERR1658398_Kenya_2000_h2
ERR1658434_Kenya_2000_h1
ERR1702561_Mozambique_2004_h2
ERR1702557_Mozambique_2004_h1
ERR1702633_Mozambique_2004_h2
ERR1702593_Mozambique_2004_h1
ERR1702595_Mozambique_2004_h1
ERR1702628_Mozambique_2004_h1
ERR1702632_Mozambique_2004_h1
ERR1702559_Mozambique_2004_h1
ERR1702560_Mozambique_2004_h2
ERR1638833_Kenya_2000_h1
ERR1702592_Mozambique_2004_h1
ERR1702631_Mozambique_2004_h1
ERR1658434_Kenya_2000_h2
ERR1658398_Kenya_2000_h1
ERR1702563_Mozambique_2004_h2
ERR1638834_Kenya_2000_h2
ERR1658334_Mali_2004_h2
ERR323479_Cameroon_2009_h1
ERR1658402_Kenya_2000_h1
ERR1658372_Mali_2004_h1
ERR332002_Burkina_Faso_2012_h1
ERR332014_Burkina_Faso_2012_h1
ERR317297_Cameroon_2009_h2
ERR1638807_Mali_2004_h1
ERR1620638_DRCongo_2015_h2
ERR1638805_Mali_2004_h1
ERR984171_Gambia_2012_h2
ERR317374_Cameroon_2009_h1
ERR842027_Mali_2012_h2
ERR1638844_Kenya_2000_h2
ERR1620644_DRCongo_2015_h1
ERR1638531_Tanzania_2013_h2
ERR1638524_Tanzania_2013_h1
ERR216660_Mayotte_2011_h2
ERR340905_Cameroon_2009_h2
ERR984183_Gambia_2012_h1
ERR1638495_Tanzania_2013_h2
ERR1658435_Kenya_2000_h2
ERR203735_Mayotte_2011_h1
ERR842028_Mali_2012_h2
ERR1638488_Tanzania_2013_h1
ERR842040_Mali_2012_h1
ERR320223_Kenya_2012_h2
ERR842039_Mali_2012_h2
ERR327060_Kenya_2012_h1
ERR203728_Mayotte_2011_h1
ERR216666_Mayotte_2011_h1
ERR197472_Mayotte_2011_h1
ERR1638749_Cameroon_2005_h1
ERR1702559_Mozambique_2004_h2
ERR320234_Kenya_2012_h1
ERR1702591_Mozambique_2004_h2
ERR779589_Mali_2012_h1
ERR317232_Burkina_Faso_2012_h1
ERR203736_Mayotte_2011_h2
ERR320222_Kenya_2012_h2
ERR517150_Kenya_2012_h1
ERR323774_Kenya_2012_h1
ERR197458_Mayotte_2011_h2
ERR516935_Kenya_2012_h1
ERR842027_Mali_2012_h1
ERR1702278_Cameroon_2005_h2
ERR320235_Kenya_2012_h2
ERR327048_Kenya_2012_h1
ERR216678_Mayotte_2011_h1
ERR203724_Mayotte_2011_h2
ERR779577_Mali_2012_h1
ERR203716_Mayotte_2011_h2
ERR317283_Kenya_2012_h1
ERR491347_Kenya_2012_h2
ERR1702555_Mozambique_2004_h1
ERR1658441_Kenya_2000_h2
ERR1702627_Mozambique_2004_h2
ERR317284_Kenya_2012_h2
ERR495495_Kenya_2012_h1
ERR1702631_Mozambique_2004_h2
ERR517138_Kenya_2012_h1
ERR495507_Kenya_2012_h2
ERR197464_Mayotte_2011_h1
ERR1658405_Kenya_2000_h2
ERR1638840_Kenya_2000_h2
ERR1638805_Mali_2004_h2
ERR1658334_Mali_2004_h1
ERR1658370_Mali_2004_h1
ERR381775_Gambia_2011_h2
ERR779576_Mali_2012_h1
ERR984158_Gambia_2012_h1
ERR842038_Mali_2012_h2
ERR842043_Mali_2012_h1
ERR779581_Mali_2012_h2
ERR842026_Mali_2012_h1
ERR842031_Mali_2012_h1
ERR1702595_Mozambique_2004_h2
ERR1620643_DRCongo_2015_h2
ERR1554842_Tanzania_2015_h2
ERR323479_Cameroon_2009_h2
ERR501737_Uganda_2012_h2
ERR501726_Uganda_2012_h1
ERR328816_Uganda_2012_h1
ERR331975_Uganda_2012_h1
ERR327351_Uganda_2012_h2
ERR327351_Uganda_2012_h1
ERR1554984_Tanzania_2015_h2
ERR501738_Uganda_2012_h1
ERR317374_Cameroon_2009_h2
ERR1554878_Tanzania_2015_h2
ERR323491_Cameroon_2009_h2
ERR495549_Uganda_2012_h2
ERR501725_Uganda_2012_h2
ERR331987_Uganda_2012_h2
ERR1620787_DRCongo_2015_h1
ERR495548_Uganda_2012_h1
ERR1554914_Tanzania_2015_h2
ERR1554948_Tanzania_2015_h1
ERR1555020_Tanzania_2015_h2
ERR197457_Equatorial_Guinea_2002_h1
ERR984152_Gambia_2012_h1
ERR381811_Gambia_2011_h2
ERR381799_Gambia_2011_h2
ERR376473_Gambia_2011_h2
ERR516918_Guinea-Bissau_2010_h1
ERR517061_Guinea-Bissau_2010_h2
ERR517049_Guinea-Bissau_2010_h2
ERR323862_Guinea-Bissau_2010_h1
ERR328615_Guinea-Bissau_2010_h1
ERR328627_Guinea-Bissau_2010_h1
ERR517143_Guinea-Bissau_2010_h2
ERR517025_Guinea-Bissau_2010_h2
ERR984162_Gambia_2012_h2
ERR984150_Gambia_2012_h2
ERR516906_Guinea-Bissau_2010_h2
ERR517037_Guinea-Bissau_2010_h2
ERR517155_Guinea-Bissau_2010_h2
ERR984164_Gambia_2012_h1
ERR984152_Gambia_2012_h2
ERR376464_Gambia_2011_h1
ERR381778_Gambia_2011_h1
ERR970979_Gambia_2012_h2
ERR381790_Gambia_2011_h2
ERR381776_Gambia_2011_h2
ERR376462_Gambia_2011_h1
ERR381788_Gambia_2011_h1
ERR516935_Kenya_2012_h2
ERR517150_Kenya_2012_h2
ERR327048_Kenya_2012_h2

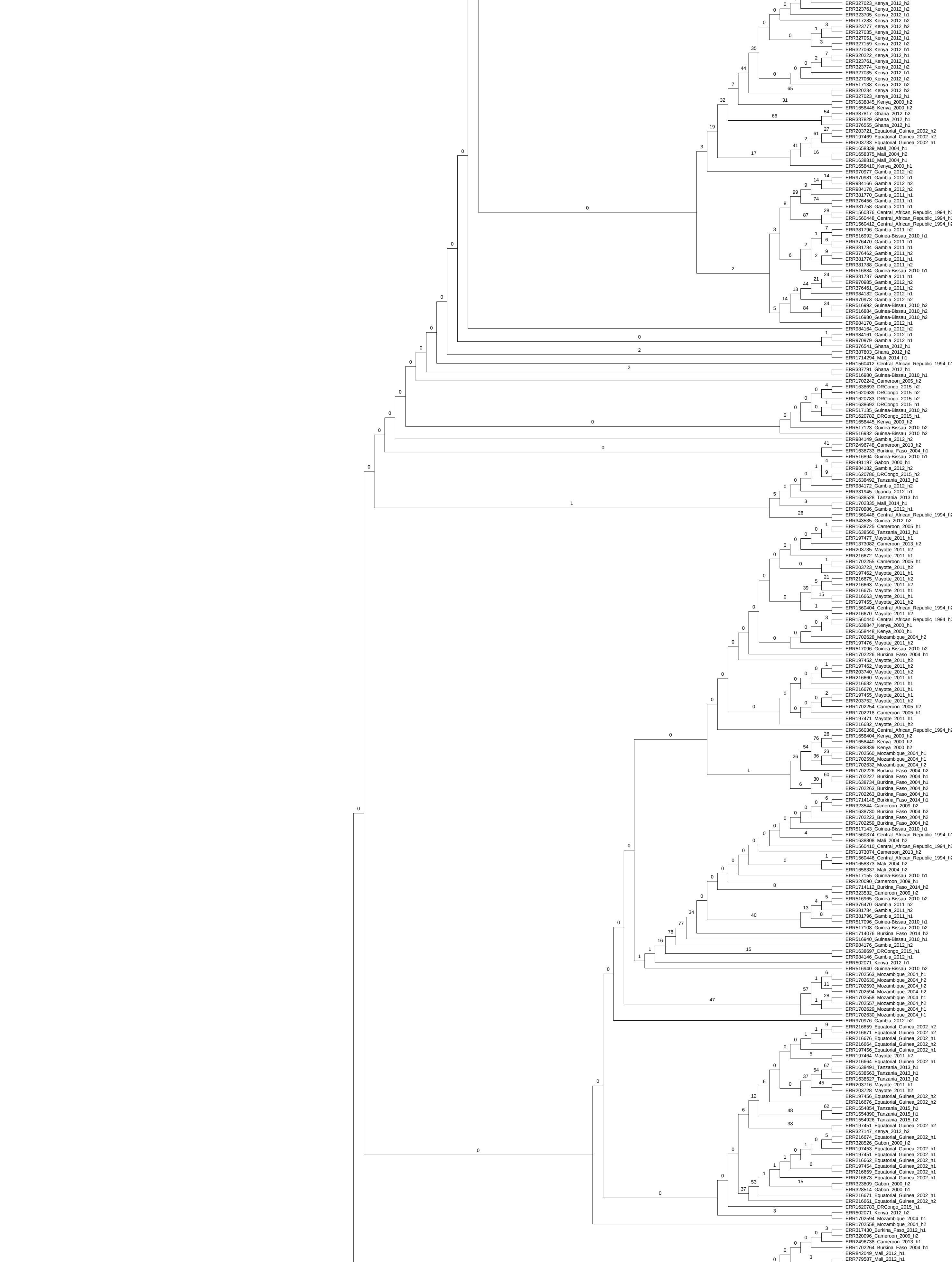
ERR327023_Kenya_2012_h2
ERR323761_Kenya_2012_h2
ERR323705_Kenya_2012_h1
ERR317283_Kenya_2012_h2
ERR323777_Kenya_2012_h2
ERR327035_Kenya_2012_h2
ERR327051_Kenya_2012_h1
ERR327159_Kenya_2012_h2
ERR327063_Kenya_2012_h1
ERR320222_Kenya_2012_h1
ERR323761_Kenya_2012_h1
ERR323774_Kenya_2012_h2
ERR327035_Kenya_2012_h1
ERR327060_Kenya_2012_h2
ERR517138_Kenya_2012_h2
ERR320234_Kenya_2012_h2
ERR327023_Kenya_2012_h1
ERR1638845_Kenya_2000_h2
ERR1658446_Kenya_2000_h2
ERR387817_Ghana_2012_h2
ERR387829_Ghana_2012_h1
ERR376555_Ghana_2012_h1
ERR203721_Equatorial_Guinea_2002_h2
ERR197469_Equatorial_Guinea_2002_h2
ERR203733_Equatorial_Guinea_2002_h1
ERR1658339_Mali_2004_h1
ERR1658375_Mali_2004_h2
ERR1638810_Mali_2004_h1
ERR1658410_Kenya_2000_h1
ERR970977_Gambia_2012_h2
ERR970981_Gambia_2012_h1
ERR984166_Gambia_2012_h2
ERR984178_Gambia_2012_h2
ERR381770_Gambia_2011_h1
ERR376456_Gambia_2011_h1
ERR381758_Gambia_2011_h1
ERR1560376_Central_African_Republic_1994_h
ERR1560448_Central_African_Republic_1994_h
ERR1560412_Central_African_Republic_1994_h
ERR381796_Gambia_2011_h2
ERR516992_Guinea-Bissau_2010_h1
ERR376470_Gambia_2011_h1
ERR381784_Gambia_2011_h1
ERR376462_Gambia_2011_h2
ERR381776_Gambia_2011_h1
ERR381788_Gambia_2011_h2
ERR516884_Guinea-Bissau_2010_h1
ERR381787_Gambia_2011_h1
ERR970985_Gambia_2012_h2
ERR376461_Gambia_2011_h2
ERR984182_Gambia_2012_h1
ERR970973_Gambia_2012_h2
ERR516992_Guinea-Bissau_2010_h2
ERR516884_Guinea-Bissau_2010_h2
ERR516980_Guinea-Bissau_2010_h2
ERR984170_Gambia_2012_h1
ERR984164_Gambia_2012_h2
ERR984161_Gambia_2012_h1
ERR970979_Gambia_2012_h1
ERR376541_Ghana_2012_h1
ERR387803_Ghana_2012_h2
ERR1714294_Mali_2014_h1
ERR1560412_Central_African_Republic_1994_h
ERR387791_Ghana_2012_h1
ERR516980_Guinea-Bissau_2010_h1
ERR1702242_Cameroon_2005_h2
ERR1638693_DRCongo_2015_h2
ERR1620639_DRCongo_2015_h2
ERR1620783_DRCongo_2015_h2
ERR1638692_DRCongo_2015_h1
ERR517135_Guinea-Bissau_2010_h2
ERR1620782_DRCongo_2015_h1
ERR1658445_Kenya_2000_h2
ERR517123_Guinea-Bissau_2010_h2
ERR516932_Guinea-Bissau_2010_h2
ERR984149_Gambia_2012_h2
ERR2496748_Cameroon_2013_h2
ERR1638733_Burkina_Faso_2004_h1
ERR516894_Guinea-Bissau_2010_h1
ERR491197_Gabon_2000_h1
ERR984182_Gambia_2012_h2
ERR1620786_DRCongo_2015_h2
ERR1638492_Tanzania_2013_h2
ERR984172_Gambia_2012_h2
ERR331945_Uganda_2012_h1
ERR1638528_Tanzania_2013_h1
ERR1702335_Mali_2014_h1
ERR970986_Gambia_2012_h1
ERR1560448_Central_African_Republic_1994_h
ERR343535_Guinea_2012_h2
ERR1638725_Cameroon_2005_h1
ERR1638560_Tanzania_2013_h1
ERR197477_Mayotte_2011_h1
ERR1373082_Cameroon_2013_h2
ERR203735_Mayotte_2011_h2
ERR216672_Mayotte_2011_h1
ERR1702255_Cameroon_2005_h1
ERR203723_Mayotte_2011_h2
ERR197462_Mayotte_2011_h1
ERR216675_Mayotte_2011_h2
ERR216663_Mayotte_2011_h2
ERR216675_Mayotte_2011_h1
ERR216663_Mayotte_2011_h1
ERR197455_Mayotte_2011_h2
ERR1560404_Central_African_Republic_1994_h
ERR216670_Mayotte_2011_h2
ERR1560440_Central_African_Republic_1994_h
ERR1638847_Kenya_2000_h1
ERR1658448_Kenya_2000_h1
ERR1702628_Mozambique_2004_h2
ERR197476_Mayotte_2011_h2
ERR517096_Guinea-Bissau_2010_h2
ERR1702226_Burkina_Faso_2004_h1
ERR197452_Mayotte_2011_h2
ERR197462_Mayotte_2011_h2
ERR203740_Mayotte_2011_h2
ERR216660_Mayotte_2011_h1
ERR216682_Mayotte_2011_h1
ERR216670_Mayotte_2011_h1
ERR197455_Mayotte_2011_h1
ERR203752_Mayotte_2011_h2
ERR1702254_Cameroon_2005_h2
ERR1702218_Cameroon_2005_h1
ERR197471_Mayotte_2011_h1
ERR216682_Mayotte_2011_h2
ERR1560368_Central_African_Republic_1994_h
ERR1658404_Kenya_2000_h2
ERR1658440_Kenya_2000_h2
ERR1638839_Kenya_2000_h2
ERR1702560_Mozambique_2004_h1
ERR1702596_Mozambique_2004_h1
ERR1702632_Mozambique_2004_h2
ERR1702226_Burkina_Faso_2004_h2
ERR1702227_Burkina_Faso_2004_h1
ERR1638734_Burkina_Faso_2004_h1
ERR1702263_Burkina_Faso_2004_h2
ERR1702263_Burkina_Faso_2004_h1
ERR1714148_Burkina_Faso_2014_h1
ERR323544_Cameroon_2009_h2
ERR1638730_Burkina_Faso_2004_h2
ERR1702223_Burkina_Faso_2004_h2
ERR1702259_Burkina_Faso_2004_h2
ERR517143_Guinea-Bissau_2010_h1
ERR1560374_Central_African_Republic_1994_h
ERR1638808_Mali_2004_h2
ERR1560410_Central_African_Republic_1994_h
ERR1373074_Cameroon_2013_h2
ERR1560446_Central_African_Republic_1994_h
ERR1658373_Mali_2004_h2
ERR1658337_Mali_2004_h2
ERR517155_Guinea-Bissau_2010_h1
ERR320090_Cameroon_2009_h1
ERR1714112_Burkina_Faso_2014_h2
ERR323532_Cameroon_2009_h2
ERR516965_Guinea-Bissau_2010_h2
ERR376470_Gambia_2011_h2
ERR381784_Gambia_2011_h2
ERR381796_Gambia_2011_h1
ERR517096_Guinea-Bissau_2010_h1
ERR517108_Guinea-Bissau_2010_h2
ERR1714076_Burkina_Faso_2014_h2
ERR516940_Guinea-Bissau_2010_h1
ERR984176_Gambia_2012_h2
ERR1638697_DRCongo_2015_h1
ERR984146_Gambia_2012_h1
ERR502071_Kenya_2012_h1
ERR516940_Guinea-Bissau_2010_h2
ERR1702563_Mozambique_2004_h1
ERR1702630_Mozambique_2004_h2
ERR1702593_Mozambique_2004_h2
ERR1702594_Mozambique_2004_h2
ERR1702558_Mozambique_2004_h1
ERR1702557_Mozambique_2004_h2
ERR1702629_Mozambique_2004_h1
ERR1702630_Mozambique_2004_h1
ERR970976_Gambia_2012_h2
ERR216659_Equatorial_Guinea_2002_h2
ERR216671_Equatorial_Guinea_2002_h2
ERR216676_Equatorial_Guinea_2002_h1
ERR216664_Equatorial_Guinea_2002_h2
ERR197456_Equatorial_Guinea_2002_h1
ERR197464_Mayotte_2011_h2
ERR216664_Equatorial_Guinea_2002_h1
ERR1638491_Tanzania_2013_h1
ERR1638563_Tanzania_2013_h1
ERR1638527_Tanzania_2013_h2
ERR203716_Mayotte_2011_h1
ERR203728_Mayotte_2011_h2
ERR197456_Equatorial_Guinea_2002_h2
ERR216676_Equatorial_Guinea_2002_h2
ERR1554854_Tanzania_2015_h1
ERR1554890_Tanzania_2015_h1
ERR1554926_Tanzania_2015_h2
ERR197451_Equatorial_Guinea_2002_h2
ERR327147_Kenya_2012_h2
ERR216674_Equatorial_Guinea_2002_h1
ERR328526_Gabon_2000_h2
ERR197453_Equatorial_Guinea_2002_h1
ERR197451_Equatorial_Guinea_2002_h1
ERR216662_Equatorial_Guinea_2002_h1
ERR197454_Equatorial_Guinea_2002_h1
ERR216659_Equatorial_Guinea_2002_h1
ERR216673_Equatorial_Guinea_2002_h1
ERR323809_Gabon_2000_h2
ERR328514_Gabon_2000_h1
ERR216671_Equatorial_Guinea_2002_h1
ERR216661_Equatorial_Guinea_2002_h2
ERR1620783_DRCongo_2015_h1
ERR502071_Kenya_2012_h2
ERR1702594_Mozambique_2004_h1
ERR1702558_Mozambique_2004_h2
ERR317430_Burkina_Faso_2012_h1
ERR320096_Cameroon_2009_h2
ERR2496738_Cameroon_2013_h1
ERR1702264_Burkina_Faso_2004_h1
ERR842049_Mali_2012_h1
ERR779587_Mali_2012_h1

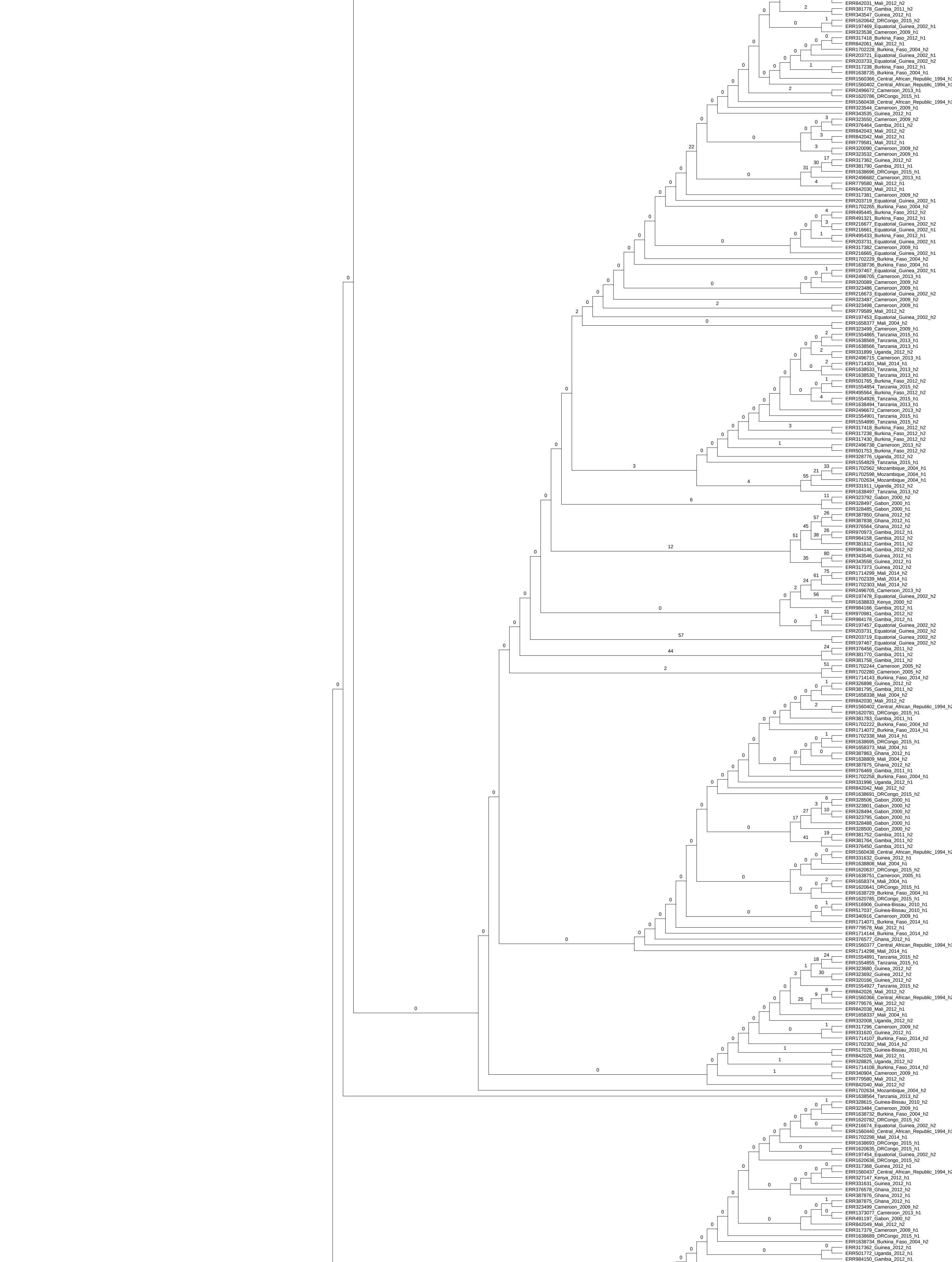
ERR842031_Mali_2012_h2
ERR381778_Gambia_2011_h2
ERR343547_Guinea_2012_h1
ERR1620642_DRCongo_2015_h2
ERR197469_Equatorial_Guinea_2002_h1
ERR323538_Cameroon_2009_h1
ERR317418_Burkina_Faso_2012_h1
ERR842061_Mali_2012_h1
ERR1702228_Burkina_Faso_2004_h2
ERR203721_Equatorial_Guinea_2002_h1
ERR203733_Equatorial_Guinea_2002_h2
ERR317238_Burkina_Faso_2012_h1
ERR1638735_Burkina_Faso_2004_h1
ERR1560366_Central_African_Republic_1994_h1
ERR1560402_Central_African_Republic_1994_h1
ERR2496672_Cameroon_2013_h1
ERR1620786_DRCongo_2015_h1
ERR1560438_Central_African_Republic_1994_h1
ERR323544_Cameroon_2009_h1
ERR343535_Guinea_2012_h1
ERR323550_Cameroon_2009_h2
ERR376464_Gambia_2011_h2
ERR842043_Mali_2012_h2
ERR842042_Mali_2012_h1
ERR779581_Mali_2012_h1
ERR320090_Cameroon_2009_h2
ERR323532_Cameroon_2009_h1
ERR317362_Guinea_2012_h2
ERR381790_Gambia_2011_h1
ERR1638696_DRCongo_2015_h1
ERR2496682_Cameroon_2013_h1
ERR779580_Mali_2012_h1
ERR842030_Mali_2012_h1
ERR317381_Cameroon_2009_h2
ERR203719_Equatorial_Guinea_2002_h1
ERR1702265_Burkina_Faso_2004_h2
ERR495445_Burkina_Faso_2012_h2
ERR491321_Burkina_Faso_2012_h1
ERR216677_Equatorial_Guinea_2002_h2
ERR216661_Equatorial_Guinea_2002_h1
ERR495433_Burkina_Faso_2012_h1
ERR203731_Equatorial_Guinea_2002_h1
ERR317382_Cameroon_2009_h1
ERR216665_Equatorial_Guinea_2002_h1
ERR1702229_Burkina_Faso_2004_h2
ERR1638736_Burkina_Faso_2004_h1
ERR197467_Equatorial_Guinea_2002_h1
ERR2496705_Cameroon_2013_h1
ERR320089_Cameroon_2009_h2
ERR323486_Cameroon_2009_h1
ERR216673_Equatorial_Guinea_2002_h2
ERR323487_Cameroon_2009_h2
ERR323498_Cameroon_2009_h1
ERR779589_Mali_2012_h2
ERR197453_Equatorial_Guinea_2002_h2
ERR1658377_Mali_2004_h2
ERR323499_Cameroon_2009_h1
ERR1554865_Tanzania_2015_h1
ERR1638569_Tanzania_2013_h1
ERR1638566_Tanzania_2013_h1
ERR331899_Uganda_2012_h2
ERR2496715_Cameroon_2013_h1
ERR1714301_Mali_2014_h1
ERR1638533_Tanzania_2013_h2
ERR1638530_Tanzania_2013_h1
ERR501765_Burkina_Faso_2012_h2
ERR1554854_Tanzania_2015_h2
ERR495564_Burkina_Faso_2012_h2
ERR1554926_Tanzania_2015_h1
ERR1638494_Tanzania_2013_h1
ERR2496672_Cameroon_2013_h2
ERR1554901_Tanzania_2015_h1
ERR1554890_Tanzania_2015_h2
ERR317418_Burkina_Faso_2012_h2
ERR317238_Burkina_Faso_2012_h2
ERR317430_Burkina_Faso_2012_h2
ERR2496738_Cameroon_2013_h2
ERR501753_Burkina_Faso_2012_h2
ERR328776_Uganda_2012_h2
ERR1554829_Tanzania_2015_h1
ERR1702562_Mozambique_2004_h1
ERR1702598_Mozambique_2004_h1
ERR1702634_Mozambique_2004_h1
ERR331911_Uganda_2012_h2
ERR1638497_Tanzania_2013_h2
ERR323792_Gabon_2000_h2
ERR328497_Gabon_2000_h1
ERR328485_Gabon_2000_h1
ERR387850_Ghana_2012_h2
ERR387838_Ghana_2012_h1
ERR376564_Ghana_2012_h2
ERR970973_Gambia_2012_h1
ERR984158_Gambia_2012_h2
ERR381812_Gambia_2011_h2
ERR984146_Gambia_2012_h2
ERR343546_Guinea_2012_h1
ERR343558_Guinea_2012_h1
ERR317373_Guinea_2012_h2
ERR1714299_Mali_2014_h2
ERR1702339_Mali_2014_h1
ERR1702303_Mali_2014_h2
ERR2496705_Cameroon_2013_h2
ERR197478_Equatorial_Guinea_2002_h2
ERR1638833_Kenya_2000_h2
ERR984166_Gambia_2012_h1
ERR970981_Gambia_2012_h2
ERR984178_Gambia_2012_h1
ERR197457_Equatorial_Guinea_2002_h2
ERR203731_Equatorial_Guinea_2002_h2
ERR203719_Equatorial_Guinea_2002_h2
ERR197467_Equatorial_Guinea_2002_h2
ERR376456_Gambia_2011_h2
ERR381770_Gambia_2011_h2
ERR381758_Gambia_2011_h2
ERR1702244_Cameroon_2005_h2
ERR1702280_Cameroon_2005_h2
ERR1714143_Burkina_Faso_2014_h2
ERR326898_Guinea_2012_h2
ERR381795_Gambia_2011_h2
ERR1658338_Mali_2004_h2
ERR842030_Mali_2012_h2
ERR1560402_Central_African_Republic_1994_h2
ERR1620781_DRCongo_2015_h1
ERR381783_Gambia_2011_h1
ERR1702222_Burkina_Faso_2004_h2
ERR1714072_Burkina_Faso_2014_h1
ERR1702338_Mali_2014_h1
ERR1638695_DRCongo_2015_h1
ERR1658373_Mali_2004_h1
ERR387863_Ghana_2012_h1
ERR1638809_Mali_2004_h2
ERR387875_Ghana_2012_h2
ERR376469_Gambia_2011_h1
ERR1702258_Burkina_Faso_2004_h1
ERR331996_Uganda_2012_h1
ERR842042_Mali_2012_h2
ERR1638691_DRCongo_2015_h2
ERR328506_Gabon_2000_h1
ERR323801_Gabon_2000_h2
ERR328494_Gabon_2000_h2
ERR323795_Gabon_2000_h1
ERR328488_Gabon_2000_h1
ERR328500_Gabon_2000_h2
ERR381752_Gambia_2011_h2
ERR381764_Gambia_2011_h2
ERR376450_Gambia_2011_h2
ERR1560438_Central_African_Republic_1994_h2
ERR331632_Guinea_2012_h1
ERR1638808_Mali_2004_h1
ERR1620637_DRCongo_2015_h2
ERR1638751_Cameroon_2005_h1
ERR1658374_Mali_2004_h1
ERR1620641_DRCongo_2015_h1
ERR1638729_Burkina_Faso_2004_h1
ERR1620785_DRCongo_2015_h1
ERR516906_Guinea-Bissau_2010_h1
ERR517037_Guinea-Bissau_2010_h1
ERR340916_Cameroon_2009_h1
ERR1714071_Burkina_Faso_2014_h1
ERR779578_Mali_2012_h1
ERR1714144_Burkina_Faso_2014_h2
ERR376577_Ghana_2012_h1
ERR1560377_Central_African_Republic_1994_h1
ERR1714298_Mali_2014_h1
ERR1554891_Tanzania_2015_h2
ERR1554855_Tanzania_2015_h1
ERR323680_Guinea_2012_h2
ERR323692_Guinea_2012_h2
ERR320166_Guinea_2012_h2
ERR1554927_Tanzania_2015_h2
ERR842026_Mali_2012_h2
ERR1560366_Central_African_Republic_1994_h2
ERR779576_Mali_2012_h2
ERR842038_Mali_2012_h1
ERR1658337_Mali_2004_h1
ERR332008_Uganda_2012_h2
ERR317296_Cameroon_2009_h2
ERR331620_Guinea_2012_h1
ERR1714107_Burkina_Faso_2014_h2
ERR1702302_Mali_2014_h2
ERR517025_Guinea-Bissau_2010_h1
ERR842028_Mali_2012_h1
ERR328825_Uganda_2012_h2
ERR1714108_Burkina_Faso_2014_h2
ERR340904_Cameroon_2009_h1
ERR779580_Mali_2012_h2
ERR842040_Mali_2012_h2
ERR1702634_Mozambique_2004_h2
ERR1638564_Tanzania_2013_h2
ERR328615_Guinea-Bissau_2010_h2
ERR323484_Cameroon_2009_h1
ERR1638732_Burkina_Faso_2004_h2
ERR1620782_DRCongo_2015_h2
ERR216674_Equatorial_Guinea_2002_h2
ERR1560440_Central_African_Republic_1994_h1
ERR1702298_Mali_2014_h1
ERR1638693_DRCongo_2015_h1
ERR1620635_DRCongo_2015_h1
ERR197454_Equatorial_Guinea_2002_h2
ERR1620636_DRCongo_2015_h2
ERR317368_Guinea_2012_h1
ERR1560437_Central_African_Republic_1994_h2
ERR327147_Kenya_2012_h1
ERR331631_Guinea_2012_h1
ERR376578_Ghana_2012_h2
ERR387876_Ghana_2012_h1
ERR387875_Ghana_2012_h1
ERR323499_Cameroon_2009_h2
ERR1373077_Cameroon_2013_h1
ERR491197_Gabon_2000_h2
ERR842049_Mali_2012_h2
ERR317379_Cameroon_2009_h1
ERR1638689_DRCongo_2015_h1
ERR1638734_Burkina_Faso_2004_h2
ERR317362_Guinea_2012_h1
ERR501772_Uganda_2012_h1
ERR984150_Gambia_2012_h1

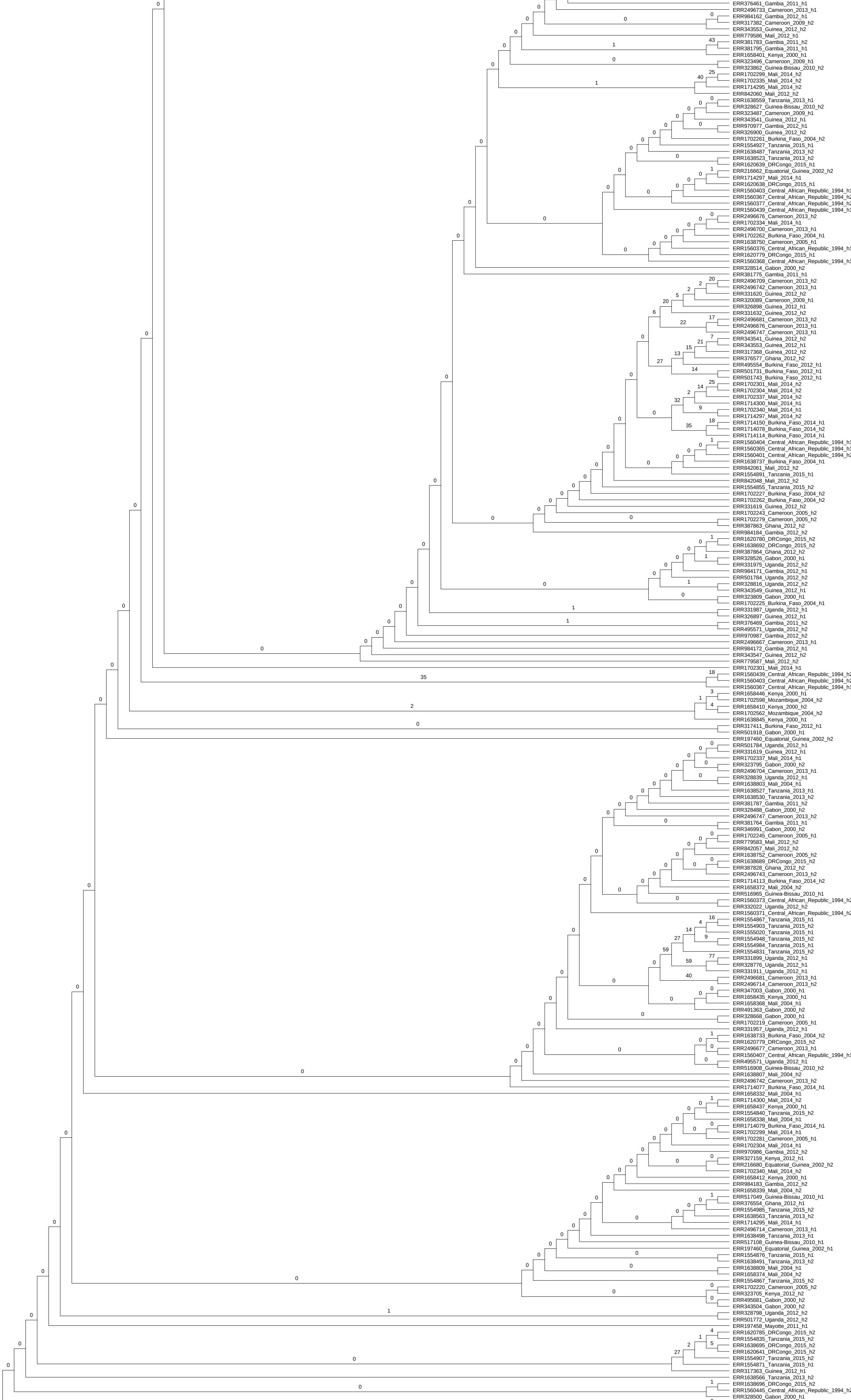

ERR376461_Gambia_2011_h1
ERR2496733_Cameroon_2013_h1
ERR984162_Gambia_2012_h1
ERR317382_Cameroon_2009_h2
ERR343553_Guinea_2012_h2
ERR779586_Mali_2012_h1
ERR381783_Gambia_2011_h2
ERR381795_Gambia_2011_h1
ERR1658401_Kenya_2000_h1
ERR323496_Cameroon_2009_h1
ERR323862_Guinea-Bissau_2010_h2
ERR1702299_Mali_2014_h2
ERR1702335_Mali_2014_h2
ERR1714295_Mali_2014_h2
ERR842060_Mali_2012_h2
ERR1638559_Tanzania_2013_h1
ERR328627_Guinea-Bissau_2010_h2
ERR323487_Cameroon_2009_h1
ERR343541_Guinea_2012_h1
ERR970977_Gambia_2012_h1
ERR326900_Guinea_2012_h2
ERR1702261_Burkina_Faso_2004_h2
ERR1554927_Tanzania_2015_h1
ERR1638487_Tanzania_2013_h2
ERR1638523_Tanzania_2013_h2
ERR1620639_DRCongo_2015_h1
ERR216662_Equatorial_Guinea_2002_h2
ERR1714297_Mali_2014_h1
ERR1620638_DRCongo_2015_h1
ERR1560403_Central_African_Republic_1994_h1
ERR1560367_Central_African_Republic_1994_h2
ERR1560377_Central_African_Republic_1994_h2
ERR1560439_Central_African_Republic_1994_h1
ERR2496676_Cameroon_2013_h2
ERR1702334_Mali_2014_h1
ERR2496700_Cameroon_2013_h1
ERR1702262_Burkina_Faso_2004_h1
ERR1638750_Cameroon_2005_h1
ERR1560376_Central_African_Republic_1994_h1
ERR1620779_DRCongo_2015_h1
ERR1560368_Central_African_Republic_1994_h1
ERR328514_Gabon_2000_h2
ERR381775_Gambia_2011_h1
ERR2496709_Cameroon_2013_h2
ERR2496742_Cameroon_2013_h1
ERR331620_Guinea_2012_h2
ERR320089_Cameroon_2009_h1
ERR326898_Guinea_2012_h1
ERR331632_Guinea_2012_h2
ERR2496681_Cameroon_2013_h2
ERR2496676_Cameroon_2013_h1
ERR2496747_Cameroon_2013_h1
ERR343541_Guinea_2012_h2
ERR343553_Guinea_2012_h1
ERR317368_Guinea_2012_h2
ERR376577_Ghana_2012_h2
ERR495554_Burkina_Faso_2012_h1
ERR501731_Burkina_Faso_2012_h1
ERR501743_Burkina_Faso_2012_h1
ERR1702301_Mali_2014_h2
ERR1702304_Mali_2014_h2
ERR1702337_Mali_2014_h2
ERR1714300_Mali_2014_h1
ERR1702340_Mali_2014_h1
ERR1714297_Mali_2014_h2
ERR1714150_Burkina_Faso_2014_h1
ERR1714078_Burkina_Faso_2014_h2
ERR1714114_Burkina_Faso_2014_h1
ERR1560404_Central_African_Republic_1994_h1
ERR1560365_Central_African_Republic_1994_h1
ERR1560401_Central_African_Republic_1994_h2
ERR1638737_Burkina_Faso_2004_h1
ERR842061_Mali_2012_h2
ERR1554891_Tanzania_2015_h1
ERR842048_Mali_2012_h2
ERR1554855_Tanzania_2015_h2
ERR1702227_Burkina_Faso_2004_h2
ERR1702262_Burkina_Faso_2004_h2
ERR331619_Guinea_2012_h2
ERR1702243_Cameroon_2005_h2
ERR1702279_Cameroon_2005_h2
ERR387863_Ghana_2012_h2
ERR984184_Gambia_2012_h2
ERR1620780_DRCongo_2015_h2
ERR1638692_DRCongo_2015_h2
ERR387864_Ghana_2012_h2
ERR328526_Gabon_2000_h1
ERR331975_Uganda_2012_h2
ERR984171_Gambia_2012_h1
ERR501784_Uganda_2012_h2
ERR328816_Uganda_2012_h2
ERR343549_Guinea_2012_h1
ERR323809_Gabon_2000_h1
ERR1702225_Burkina_Faso_2004_h1
ERR331987_Uganda_2012_h1
ERR326897_Guinea_2012_h1
ERR376469_Gambia_2011_h2
ERR495571_Uganda_2012_h2
ERR970987_Gambia_2012_h2
ERR2496667_Cameroon_2013_h1
ERR984172_Gambia_2012_h1
ERR343547_Guinea_2012_h2
ERR779587_Mali_2012_h2
ERR1702301_Mali_2014_h1
ERR1560439_Central_African_Republic_1994_h2
ERR1560403_Central_African_Republic_1994_h2
ERR1560367_Central_African_Republic_1994_h1
ERR1658446_Kenya_2000_h1
ERR1702598_Mozambique_2004_h2
ERR1658410_Kenya_2000_h2
ERR1702562_Mozambique_2004_h2
ERR1638845_Kenya_2000_h1
ERR317411_Burkina_Faso_2012_h1
ERR501918_Gabon_2000_h1
ERR197460_Equatorial_Guinea_2002_h2
ERR501784_Uganda_2012_h1
ERR331619_Guinea_2012_h1
ERR1702337_Mali_2014_h1
ERR323795_Gabon_2000_h2
ERR2496704_Cameroon_2013_h1
ERR328839_Uganda_2012_h1
ERR1638803_Mali_2004_h1
ERR1638527_Tanzania_2013_h1
ERR1638530_Tanzania_2013_h2
ERR381787_Gambia_2011_h2
ERR328488_Gabon_2000_h2
ERR2496747_Cameroon_2013_h2
ERR381764_Gambia_2011_h1
ERR346991_Gabon_2000_h2
ERR1702245_Cameroon_2005_h1
ERR779583_Mali_2012_h2
ERR842057_Mali_2012_h2
ERR1638752_Cameroon_2005_h2
ERR1638689_DRCongo_2015_h2
ERR387828_Ghana_2012_h2
ERR2496743_Cameroon_2013_h2
ERR1714113_Burkina_Faso_2014_h2
ERR1658372_Mali_2004_h2
ERR516965_Guinea-Bissau_2010_h1
ERR1560373_Central_African_Republic_1994_h2
ERR332022_Uganda_2012_h2
ERR1560371_Central_African_Republic_1994_h2
ERR1554867_Tanzania_2015_h1
ERR1554903_Tanzania_2015_h2
ERR1555020_Tanzania_2015_h1
ERR1554948_Tanzania_2015_h2
ERR1554984_Tanzania_2015_h1
ERR1554831_Tanzania_2015_h2
ERR331899_Uganda_2012_h1
ERR328776_Uganda_2012_h1
ERR331911_Uganda_2012_h1
ERR2496681_Cameroon_2013_h1
ERR2496714_Cameroon_2013_h2
ERR347003_Gabon_2000_h1
ERR1658435_Kenya_2000_h1
ERR1658368_Mali_2004_h1
ERR491363_Gabon_2000_h2
ERR328668_Gabon_2000_h1
ERR1702219_Cameroon_2005_h1
ERR331957_Uganda_2012_h1
ERR1638733_Burkina_Faso_2004_h2
ERR1620779_DRCongo_2015_h2
ERR2496677_Cameroon_2013_h1
ERR1560407_Central_African_Republic_1994_h1
ERR495571_Uganda_2012_h1
ERR516908_Guinea-Bissau_2010_h2
ERR1638807_Mali_2004_h2
ERR2496742_Cameroon_2013_h2
ERR1714077_Burkina_Faso_2014_h1
ERR1658332_Mali_2004_h1
ERR1714300_Mali_2014_h2
ERR1658437_Kenya_2000_h1
ERR1554840_Tanzania_2015_h2
ERR1658338_Mali_2004_h1
ERR1714079_Burkina_Faso_2014_h1
ERR1702299_Mali_2014_h1
ERR1702281_Cameroon_2005_h1
ERR1702304_Mali_2014_h1
ERR970986_Gambia_2012_h2
ERR327159_Kenya_2012_h1
ERR216680_Equatorial_Guinea_2002_h2
ERR1702340_Mali_2014_h2
ERR1658412_Kenya_2000_h1
ERR984183_Gambia_2012_h2
ERR1658339_Mali_2004_h2
ERR517049_Guinea-Bissau_2010_h1
ERR376554_Ghana_2012_h1
ERR1554985_Tanzania_2015_h2
ERR1638563_Tanzania_2013_h2
ERR1714295_Mali_2014_h1
ERR2496714_Cameroon_2013_h1
ERR1638498_Tanzania_2013_h1
ERR517108_Guinea-Bissau_2010_h1
ERR197460_Equatorial_Guinea_2002_h1
ERR1554876_Tanzania_2015_h1
ERR1638491_Tanzania_2013_h2
ERR1638809_Mali_2004_h1
ERR1658374_Mali_2004_h2
ERR1554867_Tanzania_2015_h2
ERR1702220_Cameroon_2005_h2
ERR323705_Kenya_2012_h2
ERR495681_Gabon_2000_h2
ERR343504_Gabon_2000_h2
ERR328798_Uganda_2012_h2
ERR501772_Uganda_2012_h2
ERR197458_Mayotte_2011_h1
ERR1620785_DRCongo_2015_h2
ERR1554835_Tanzania_2015_h2
ERR1638695_DRCongo_2015_h2
ERR1620641_DRCongo_2015_h2
ERR1554907_Tanzania_2015_h2
ERR1554871_Tanzania_2015_h1
ERR317363_Guinea_2012_h1
ERR1638566_Tanzania_2013_h2
ERR1638696_DRCongo_2015_h2
ERR1560445_Central_African_Republic_1994_h2
ERR328500_Gabon_2000_h1

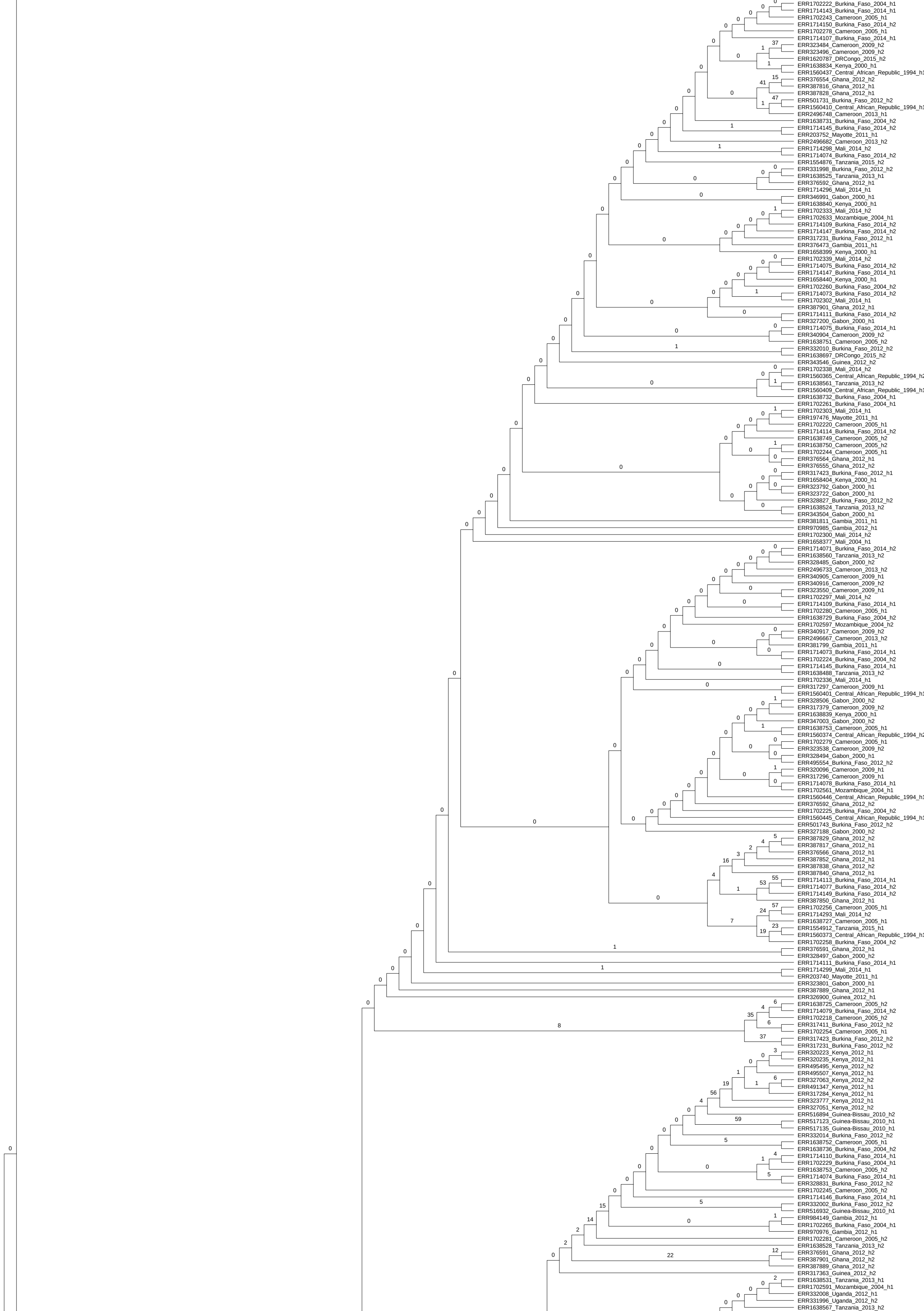
ERR1702222_Burkina_Faso_2004_h1
ERR1714143_Burkina_Faso_2014_h1
ERR1702243_Cameroon_2005_h1
ERR1714150_Burkina_Faso_2014_h2
ERR1702278_Cameroon_2005_h1
ERR1714107_Burkina_Faso_2014_h1
ERR323484_Cameroon_2009_h2
ERR323496_Cameroon_2009_h2
ERR1620787_DRCongo_2015_h2
ERR1638834_Kenya_2000_h1
ERR1560437_Central_African_Republic_1994_h1
ERR376554_Ghana_2012_h2
ERR387816_Ghana_2012_h1
ERR387828_Ghana_2012_h1
ERR501731_Burkina_Faso_2012_h2
ERR1560410_Central_African_Republic_1994_h1
ERR2496748_Cameroon_2013_h1
ERR1638731_Burkina_Faso_2004_h2
ERR1714145_Burkina_Faso_2014_h2
ERR203752_Mayotte_2011_h1
ERR2496682_Cameroon_2013_h2
ERR1714298_Mali_2014_h2
ERR1714074_Burkina_Faso_2014_h2
ERR1554876_Tanzania_2015_h2
ERR331998_Burkina_Faso_2012_h2
ERR1638525_Tanzania_2013_h1
ERR376592_Ghana_2012_h1
ERR1714296_Mali_2014_h1
ERR346991_Gabon_2000_h1
ERR1638840_Kenya_2000_h1
ERR1702333_Mali_2014_h2
ERR1702633_Mozambique_2004_h1
ERR1714109_Burkina_Faso_2014_h2
ERR1714147_Burkina_Faso_2014_h2
ERR317231_Burkina_Faso_2012_h1
ERR376473_Gambia_2011_h1
ERR1658399_Kenya_2000_h1
ERR1702339_Mali_2014_h2
ERR1714075_Burkina_Faso_2014_h2
ERR1714147_Burkina_Faso_2014_h1
ERR1658440_Kenya_2000_h1
ERR1702260_Burkina_Faso_2004_h2
ERR1714073_Burkina_Faso_2014_h2
ERR1702302_Mali_2014_h1
ERR387901_Ghana_2012_h1
ERR1714111_Burkina_Faso_2014_h2
ERR327200_Gabon_2000_h1
ERR1714075_Burkina_Faso_2014_h1
ERR340904_Cameroon_2009_h2
ERR1638751_Cameroon_2005_h2
ERR332010_Burkina_Faso_2012_h2
ERR1638697_DRCongo_2015_h2
ERR343546_Guinea_2012_h2
ERR1702338_Mali_2014_h2
ERR1560365_Central_African_Republic_1994_h2
ERR1638561_Tanzania_2013_h2
ERR1560409_Central_African_Republic_1994_h1
ERR1638732_Burkina_Faso_2004_h1
ERR1702261_Burkina_Faso_2004_h1
ERR1702303_Mali_2014_h1
ERR197476_Mayotte_2011_h1
ERR1702220_Cameroon_2005_h1
ERR1714114_Burkina_Faso_2014_h2
ERR1638749_Cameroon_2005_h2
ERR1638750_Cameroon_2005_h2
ERR1702244_Cameroon_2005_h1
ERR376564_Ghana_2012_h1
ERR376555_Ghana_2012_h2
ERR317423_Burkina_Faso_2012_h1
ERR1658404_Kenya_2000_h1
ERR323792_Gabon_2000_h1
ERR323722_Gabon_2000_h1
ERR328827_Burkina_Faso_2012_h2
ERR1638524_Tanzania_2013_h2
ERR343504_Gabon_2000_h1
ERR381811_Gambia_2011_h1
ERR970985_Gambia_2012_h1
ERR1702300_Mali_2014_h2
ERR1658377_Mali_2004_h1
ERR1714071_Burkina_Faso_2014_h2
ERR1638560_Tanzania_2013_h2
ERR328485_Gabon_2000_h2
ERR2496733_Cameroon_2013_h2
ERR340905_Cameroon_2009_h1
ERR340916_Cameroon_2009_h2
ERR323550_Cameroon_2009_h1
ERR1702297_Mali_2014_h2
ERR1714109_Burkina_Faso_2014_h1
ERR1702280_Cameroon_2005_h1
ERR1638729_Burkina_Faso_2004_h2
ERR1702597_Mozambique_2004_h2
ERR340917_Cameroon_2009_h2
ERR2496667_Cameroon_2013_h2
ERR381799_Gambia_2011_h1
ERR1714073_Burkina_Faso_2014_h1
ERR1702224_Burkina_Faso_2004_h2
ERR1714145_Burkina_Faso_2014_h1
ERR1638488_Tanzania_2013_h2
ERR1702336_Mali_2014_h1
ERR317297_Cameroon_2009_h1
ERR1560401_Central_African_Republic_1994_h1
ERR328506_Gabon_2000_h2
ERR317379_Cameroon_2009_h2
ERR1638839_Kenya_2000_h1
ERR347003_Gabon_2000_h2
ERR1638753_Cameroon_2005_h1
ERR1560374_Central_African_Republic_1994_h2
ERR1702279_Cameroon_2005_h1
ERR323538_Cameroon_2009_h2
ERR328494_Gabon_2000_h1
ERR495554_Burkina_Faso_2012_h2
ERR320096_Cameroon_2009_h1
ERR317296_Cameroon_2009_h1
ERR1714078_Burkina_Faso_2014_h1
ERR1702561_Mozambique_2004_h1
ERR1560446_Central_African_Republic_1994_h1
ERR376592_Ghana_2012_h2
ERR1702225_Burkina_Faso_2004_h2
ERR1560445_Central_African_Republic_1994_h1
ERR501743_Burkina_Faso_2012_h2
ERR327188_Gabon_2000_h2
ERR387829_Ghana_2012_h2
ERR387817_Ghana_2012_h1
ERR376566_Ghana_2012_h1
ERR387852_Ghana_2012_h1
ERR387838_Ghana_2012_h2
ERR387840_Ghana_2012_h1
ERR1714113_Burkina_Faso_2014_h1
ERR1714077_Burkina_Faso_2014_h2
ERR1714149_Burkina_Faso_2014_h2
ERR387850_Ghana_2012_h1
ERR1702256_Cameroon_2005_h1
ERR1714293_Mali_2014_h2
ERR1638727_Cameroon_2005_h1
ERR1554912_Tanzania_2015_h1
ERR1560373_Central_African_Republic_1994_h1
ERR1702258_Burkina_Faso_2004_h2
ERR376591_Ghana_2012_h1
ERR328497_Gabon_2000_h2
ERR1714111_Burkina_Faso_2014_h1
ERR1714299_Mali_2014_h1
ERR203740_Mayotte_2011_h1
ERR323801_Gabon_2000_h1
ERR387889_Ghana_2012_h1
ERR326900_Guinea_2012_h1
ERR1638725_Cameroon_2005_h2
ERR1714079_Burkina_Faso_2014_h2
ERR1702218_Cameroon_2005_h2
ERR317411_Burkina_Faso_2012_h2
ERR1702254_Cameroon_2005_h1
ERR317423_Burkina_Faso_2012_h2
ERR317231_Burkina_Faso_2012_h2
ERR320223_Kenya_2012_h1
ERR320235_Kenya_2012_h1
ERR495495_Kenya_2012_h2
ERR495507_Kenya_2012_h1
ERR327063_Kenya_2012_h2
ERR491347_Kenya_2012_h1
ERR317284_Kenya_2012_h1
ERR323777_Kenya_2012_h1
ERR327051_Kenya_2012_h2
ERR516894_Guinea-Bissau_2010_h2
ERR517123_Guinea-Bissau_2010_h1
ERR517135_Guinea-Bissau_2010_h1
ERR332014_Burkina_Faso_2012_h2
ERR1638752_Cameroon_2005_h1
ERR1638736_Burkina_Faso_2004_h2
ERR1714110_Burkina_Faso_2014_h1
ERR1702229_Burkina_Faso_2004_h1
ERR1638753_Cameroon_2005_h2
ERR1714074_Burkina_Faso_2014_h1
ERR328831_Burkina_Faso_2012_h2
ERR1702245_Cameroon_2005_h2
ERR1714146_Burkina_Faso_2014_h1
ERR332002_Burkina_Faso_2012_h2
ERR516932_Guinea-Bissau_2010_h1
ERR984149_Gambia_2012_h1
ERR1702265_Burkina_Faso_2004_h1
ERR970976_Gambia_2012_h1
ERR1702281_Cameroon_2005_h2
ERR1638528_Tanzania_2013_h2
ERR376591_Ghana_2012_h2
ERR387901_Ghana_2012_h2
ERR387889_Ghana_2012_h2
ERR317363_Guinea_2012_h2
ERR1638531_Tanzania_2013_h1
ERR1702591_Mozambique_2004_h1
ERR332008_Uganda_2012_h1
ERR331996_Uganda_2012_h2
ERR1638567_Tanzania_2013_h2

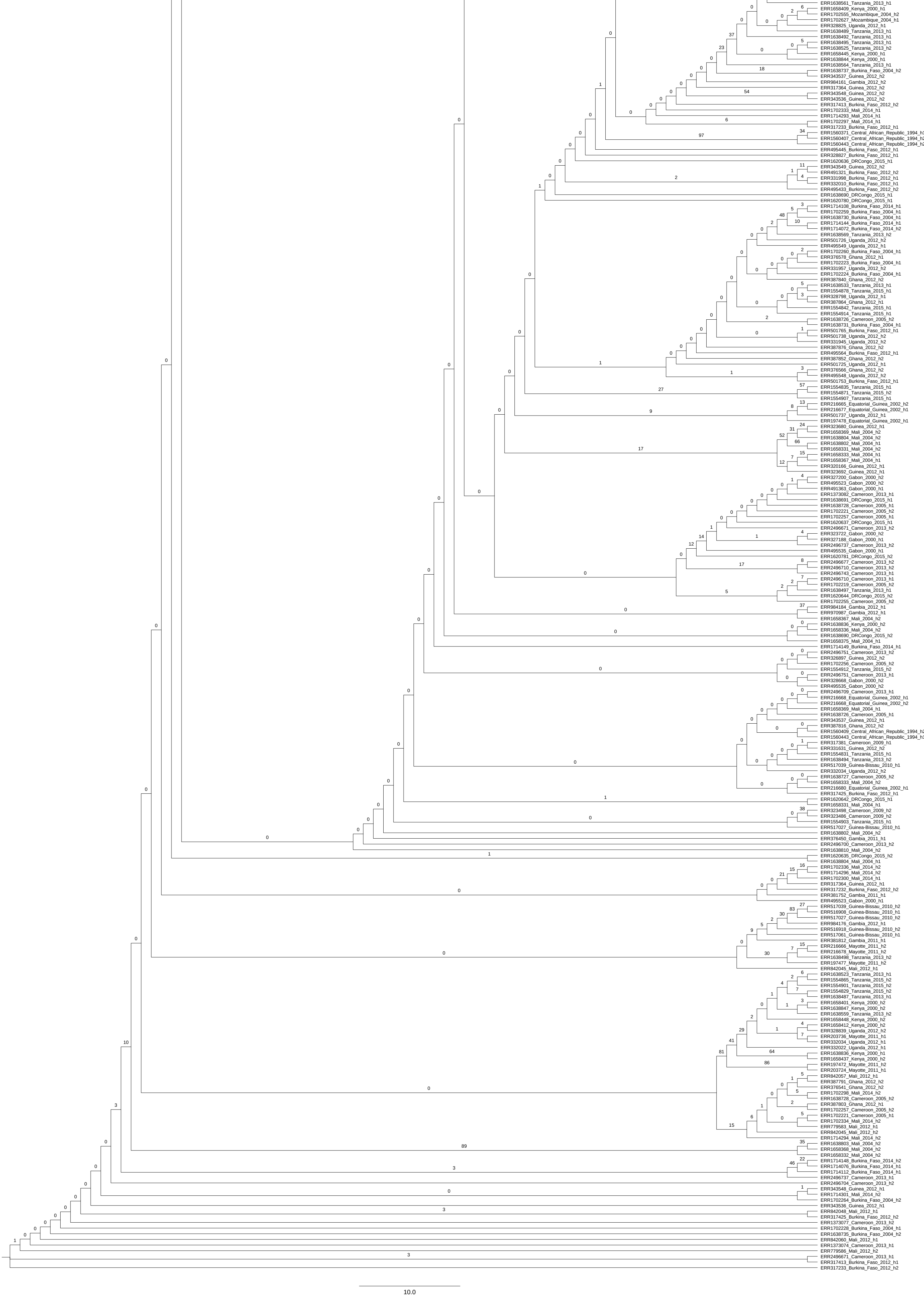
ERR1638561_Tanzania_2013_h1
ERR1658409_Kenya_2000_h1
ERR1702555_Mozambique_2004_h2
ERR1702627_Mozambique_2004_h1
ERR328825_Uganda_2012_h1
ERR1638489_Tanzania_2013_h1
ERR1638492_Tanzania_2013_h1
ERR1638495_Tanzania_2013_h1
ERR1638525_Tanzania_2013_h2
ERR1658445_Kenya_2000_h1
ERR1638844_Kenya_2000_h1
ERR1638564_Tanzania_2013_h1
ERR1638737_Burkina_Faso_2004_h2
ERR343537_Guinea_2012_h2
ERR984161_Gambia_2012_h2
ERR317364_Guinea_2012_h2
ERR343548_Guinea_2012_h2
ERR343536_Guinea_2012_h2
ERR317413_Burkina_Faso_2012_h2
ERR1702333_Mali_2014_h1
ERR1714293_Mali_2014_h1
ERR1702297_Mali_2014_h1
ERR317233_Burkina_Faso_2012_h1
ERR1560371_Central_African_Republic_1994_h1
ERR1560407_Central_African_Republic_1994_h2
ERR1560443_Central_African_Republic_1994_h2
ERR495445_Burkina_Faso_2012_h1
ERR328827_Burkina_Faso_2012_h1
ERR1620636_DRCongo_2015_h1
ERR343549_Guinea_2012_h2
ERR491321_Burkina_Faso_2012_h2
ERR331998_Burkina_Faso_2012_h1
ERR332010_Burkina_Faso_2012_h1
ERR495433_Burkina_Faso_2012_h2
ERR1638690_DRCongo_2015_h1
ERR1620780_DRCongo_2015_h1
ERR1714108_Burkina_Faso_2014_h1
ERR1702259_Burkina_Faso_2004_h1
ERR1638730_Burkina_Faso_2004_h1
ERR1714144_Burkina_Faso_2014_h1
ERR1714072_Burkina_Faso_2014_h2
ERR1638569_Tanzania_2013_h2
ERR501726_Uganda_2012_h2
ERR495549_Uganda_2012_h1
ERR1702260_Burkina_Faso_2004_h1
ERR376578_Ghana_2012_h1
ERR1702223_Burkina_Faso_2004_h1
ERR331957_Uganda_2012_h2
ERR1702224_Burkina_Faso_2004_h1
ERR387840_Ghana_2012_h2
ERR1638533_Tanzania_2013_h1
ERR1554878_Tanzania_2015_h1
ERR328798_Uganda_2012_h1
ERR387864_Ghana_2012_h1
ERR1554842_Tanzania_2015_h1
ERR1554914_Tanzania_2015_h1
ERR1638726_Cameroon_2005_h2
ERR1638731_Burkina_Faso_2004_h1
ERR501765_Burkina_Faso_2012_h1
ERR501738_Uganda_2012_h2
ERR331945_Uganda_2012_h2
ERR387876_Ghana_2012_h2
ERR495564_Burkina_Faso_2012_h1
ERR387852_Ghana_2012_h2
ERR501725_Uganda_2012_h1
ERR376566_Ghana_2012_h2
ERR495548_Uganda_2012_h2
ERR501753_Burkina_Faso_2012_h1
ERR1554835_Tanzania_2015_h1
ERR1554871_Tanzania_2015_h2
ERR1554907_Tanzania_2015_h1
ERR216665_Equatorial_Guinea_2002_h2
ERR216677_Equatorial_Guinea_2002_h1
ERR501737_Uganda_2012_h1
ERR197478_Equatorial_Guinea_2002_h1
ERR323680_Guinea_2012_h1
ERR1658369_Mali_2004_h2
ERR1638804_Mali_2004_h2
ERR1638802_Mali_2004_h1
ERR1658331_Mali_2004_h2
ERR1658333_Mali_2004_h1
ERR1658367_Mali_2004_h1
ERR320166_Guinea_2012_h1
ERR323692_Guinea_2012_h1
ERR327200_Gabon_2000_h2
ERR495523_Gabon_2000_h2
ERR491363_Gabon_2000_h1
ERR1373082_Cameroon_2013_h1
ERR1638691_DRCongo_2015_h1
ERR1638728_Cameroon_2005_h1
ERR1702221_Cameroon_2005_h2
ERR1702257_Cameroon_2005_h1
ERR1620637_DRCongo_2015_h1
ERR2496671_Cameroon_2013_h2
ERR323722_Gabon_2000_h2
ERR327188_Gabon_2000_h1
ERR2496737_Cameroon_2013_h2
ERR495535_Gabon_2000_h1
ERR1620781_DRCongo_2015_h2
ERR2496677_Cameroon_2013_h2
ERR2496710_Cameroon_2013_h2
ERR2496743_Cameroon_2013_h1
ERR2496710_Cameroon_2013_h1
ERR1702219_Cameroon_2005_h2
ERR1638497_Tanzania_2013_h1
ERR1620644_DRCongo_2015_h2
ERR1702255_Cameroon_2005_h2
ERR984184_Gambia_2012_h1
ERR970987_Gambia_2012_h1
ERR1658367_Mali_2004_h2
ERR1638836_Kenya_2000_h2
ERR1658336_Mali_2004_h2
ERR1638690_DRCongo_2015_h2
ERR1658375_Mali_2004_h1
ERR1714149_Burkina_Faso_2014_h1
ERR2496751_Cameroon_2013_h2
ERR326897_Guinea_2012_h2
ERR1702256_Cameroon_2005_h2
ERR1554912_Tanzania_2015_h2
ERR2496751_Cameroon_2013_h1
ERR328668_Gabon_2000_h2
ERR495535_Gabon_2000_h2
ERR2496709_Cameroon_2013_h1
ERR216668_Equatorial_Guinea_2002_h1
ERR216668_Equatorial_Guinea_2002_h2
ERR1658369_Mali_2004_h1
ERR1638726_Cameroon_2005_h1
ERR343537_Guinea_2012_h1
ERR387816_Ghana_2012_h2
ERR1560409_Central_African_Republic_1994_h2
ERR1560443_Central_African_Republic_1994_h1
ERR317381_Cameroon_2009_h1
ERR331631_Guinea_2012_h2
ERR1554831_Tanzania_2015_h1
ERR1638494_Tanzania_2013_h2
ERR517039_Guinea-Bissau_2010_h1
ERR332034_Uganda_2012_h2
ERR1638727_Cameroon_2005_h2
ERR1658333_Mali_2004_h2
ERR216680_Equatorial_Guinea_2002_h1
ERR317425_Burkina_Faso_2012_h1
ERR1620642_DRCongo_2015_h1
ERR1658331_Mali_2004_h1
ERR323498_Cameroon_2009_h2
ERR323486_Cameroon_2009_h2
ERR1554903_Tanzania_2015_h1
ERR517027_Guinea-Bissau_2010_h1
ERR1638802_Mali_2004_h2
ERR376450_Gambia_2011_h1
ERR2496700_Cameroon_2013_h2
ERR1638810_Mali_2004_h2
ERR1620635_DRCongo_2015_h2
ERR1638804_Mali_2004_h1
ERR1702336_Mali_2014_h2
ERR1714296_Mali_2014_h2
ERR1702300_Mali_2014_h1
ERR317364_Guinea_2012_h1
ERR317232_Burkina_Faso_2012_h2
ERR381752_Gambia_2011_h1
ERR495523_Gabon_2000_h1
ERR517039_Guinea-Bissau_2010_h2
ERR516908_Guinea-Bissau_2010_h1
ERR517027_Guinea-Bissau_2010_h2
ERR984176_Gambia_2012_h1
ERR516918_Guinea-Bissau_2010_h2
ERR517061_Guinea-Bissau_2010_h1
ERR381812_Gambia_2011_h1
ERR216666_Mayotte_2011_h2
ERR216678_Mayotte_2011_h2
ERR1638498_Tanzania_2013_h2
ERR197477_Mayotte_2011_h2
ERR842045_Mali_2012_h1
ERR1638523_Tanzania_2013_h1
ERR1554865_Tanzania_2015_h2
ERR1554901_Tanzania_2015_h2
ERR1554829_Tanzania_2015_h2
ERR1638487_Tanzania_2013_h1
ERR1658401_Kenya_2000_h2
ERR1638847_Kenya_2000_h2
ERR1638559_Tanzania_2013_h2
ERR1658448_Kenya_2000_h2
ERR1658412_Kenya_2000_h2
ERR328839_Uganda_2012_h2
ERR203736_Mayotte_2011_h1
ERR332034_Uganda_2012_h1
ERR332022_Uganda_2012_h1
ERR1638836_Kenya_2000_h1
ERR1658437_Kenya_2000_h2
ERR197472_Mayotte_2011_h2
ERR203724_Mayotte_2011_h1
ERR842057_Mali_2012_h1
ERR387791_Ghana_2012_h2
ERR376541_Ghana_2012_h2
ERR1702298_Mali_2014_h2
ERR1638728_Cameroon_2005_h2
ERR387803_Ghana_2012_h1
ERR1702257_Cameroon_2005_h2
ERR1702221_Cameroon_2005_h1
ERR1702334_Mali_2014_h2
ERR779583_Mali_2012_h1
ERR842045_Mali_2012_h2
ERR1714294_Mali_2014_h2
ERR1638803_Mali_2004_h2
ERR1658368_Mali_2004_h2
ERR1658332_Mali_2004_h2
ERR1714148_Burkina_Faso_2014_h2
ERR1714076_Burkina_Faso_2014_h1
ERR1714112_Burkina_Faso_2014_h1
ERR2496737_Cameroon_2013_h1
ERR2496704_Cameroon_2013_h2
ERR343548_Guinea_2012_h1
ERR1714301_Mali_2014_h2
ERR1702264_Burkina_Faso_2004_h2
ERR343536_Guinea_2012_h1
ERR842048_Mali_2012_h1
ERR317425_Burkina_Faso_2012_h2
ERR1373077_Cameroon_2013_h2
ERR1702228_Burkina_Faso_2004_h1
ERR1638735_Burkina_Faso_2004_h2
ERR842060_Mali_2012_h1
ERR1373074_Cameroon_2013_h1
ERR779586_Mali_2012_h2
ERR2496671_Cameroon_2013_h1
ERR317413_Burkina_Faso_2012_h1
ERR317233_Burkina_Faso_2012_h2
10.0
